# Supplementary material for: End Sequence Analysis Toolkit (ESAT) expands the extractable information from single-cell RNA-seq data
Source: Genome Res. 2016 Oct;26(10):1397–410. doi: 10.1101/gr.207902.116 (PMC5052061; doi:10.1101/gr.207902.116)
Supplement: Supplemental Material [file supp_gr.207902.116_Supp_Materials.tar.gz › ESAT-esat_v0.1/doc/html/functions_g.html]

ESAT: Class Members


ESAT


- Main Page
- Related Pages
- Packages
- Classes
- Files

- Class List
- Class Index
- Class Hierarchy
- Class Members

- All
- Functions
- Variables
- Enumerator

- a
- b
- c
- d
- e
- f
- g
- h
- i
- j
- k
- l
- m
- n
- o
- p
- q
- r
- s
- t
- u
- v
- w
- z


All Classes Namespaces Files Functions Variables Enumerator Pages

Here is a list of all class members with links to the classes they belong to:

### - g -

- G\_CODE
  : broad.core.multiplealignment.MultipleAlignment
- GAP
  : jaligner.Alignment
  , jaligner.Markups
- GAP\_CODE
  : broad.core.multiplealignment.MultipleAlignment
- gaps()
  : broad.pda.feature.genome.Chromosome
- gcContent()
  : broad.core.sequence.Sequence
- GENE
  : broad.pda.seq.rap.GenomeCommandLineProgram
- Gene()
  : umms.core.annotation.Gene
- GENE\_BED
  : broad.pda.seq.rap.rna.TranscriptomeCommandLineProgram
- geneAvgCoverage
  : broad.pda.seq.clip.SampleData
  , broad.pda.seq.protection.SampleData
  , broad.pda.seq.protection.WindowScores
- GeneCounts()
  : broad.pda.seq.segmentation.GeneCounts
- generate()
  : jaligner.matrix.MatrixGenerator
- generateAlignment()
  : broad.core.siphy.PiHMM
- generateAll5Mers()
  : broad.core.sequence.Sequence
- generateAllKmers()
  : broad.core.sequence.Sequence
- generateHKYQMatrix()
  : broad.core.siphy.EvolutionaryModelParameters
- generateRandom()
  : broad.core.datastructures.JCSCache
- generateRandomSequence()
  : broad.core.sequence.Sequence
- generateSubregions()
  : broad.pda.seq.rap.PlotAggregateRegions
- GenericAlignmentDataModel()
  : broad.pda.seq.segmentation.GenericAlignmentDataModel
- GenericRootedTree()
  : broad.core.datastructures.GenericRootedTree< T >
- genes
  : broad.pda.seq.protection.MultiSampleScanPeakCaller
  , broad.pda.seq.protection.TwoSampleScanSkellamPeakCaller
- genesByChr
  : broad.pda.seq.clip.SampleData
  , broad.pda.seq.protection.SampleData
  , broad.pda.seq.protection.WindowScores
- genesByName
  : broad.pda.seq.clip.SampleData
  , broad.pda.seq.protection.SampleData
  , broad.pda.seq.protection.WindowScores
- GeneScore()
  : broad.pda.seq.segmentation.GeneScore
- geneScores
  : broad.pda.seq.clip.SampleData
  , broad.pda.seq.protection.SampleData
  , broad.pda.seq.protection.WindowScores
- GeneSetEnrichment()
  : broad.projection.gsa.GeneSetEnrichment
- geneSpanContains()
  : umms.core.annotation.Gene
- GeneWindow()
  : umms.core.feature.GeneWindow
- GeneWithIsoforms()
  : broad.pda.gene.GeneWithIsoforms
- GENOME
  : broad.pda.samtools.PairedEndTDF
- GenomeSpaceSampleData()
  : broad.pda.seq.clip.GenomeSpaceSampleData
  , broad.pda.seq.protection.GenomeSpaceSampleData
- GenomeWindow()
  : umms.core.feature.GenomeWindow
- GenomeWithGaps()
  : broad.pda.seq.segmentation.GenomeWithGaps
- GenomeWithGaps2()
  : broad.pda.seq.segmentation.GenomeWithGaps2
- GenomicSpace()
  : umms.core.coordinatesystem.GenomicSpace
- GenomicSpanFilter()
  : nextgen.core.readFilters.GenomicSpanFilter
- genomicToGenomicPositionWithOffset()
  : umms.core.annotation.Gene
- genomicToTranscriptPosition()
  : umms.core.annotation.Gene
- geometricMean()
  : broad.core.math.Statistics
- geometricMeanMAD()
  : broad.core.math.Statistics
- get()
  : broad.core.datastructures.MatrixWithHeaders
  , broad.pda.annotation.BEDFileParser
  , Jama.Matrix
- get3PRegion()
  : broad.pda.annotation.BEDFileParser
- get3Prime()
  : broad.core.sequence.Sequence
- get3PrimeExon()
  : umms.core.annotation.Gene
- get3PrimeGenomicDistance()
  : umms.core.annotation.Gene
- get3Utr()
  : umms.core.annotation.Gene
- get3UTR()
  : umms.core.annotation.Gene
- get3UTRGene()
  : umms.core.annotation.Gene
- get3UtrIncludingIntrons()
  : umms.core.annotation.Gene
- get5PrimeExon()
  : umms.core.annotation.Gene
- get5UTR()
  : umms.core.annotation.Gene
- get5Utr()
  : umms.core.annotation.Gene
- get5UTRGene()
  : umms.core.annotation.Gene
- get5UtrIncludingIntrons()
  : umms.core.annotation.Gene
- getA()
  : broad.core.annotation.BasicTwoSubjectAnnotation
  , broad.core.annotation.TwoSubjectAnnotation
- getAbsFDR()
  : broad.core.math.FDRDistribution
- getAbsFDRMatrix()
  : broad.pda.differentialExpression.DifferentialExpression
- getAbsoluteEnd()
  : broad.core.sequence.SequenceRegion
- getAbsoluteStart()
  : broad.core.sequence.SequenceRegion
- getAEnd()
  : broad.core.annotation.BasicTwoSubjectAnnotation
  , broad.core.annotation.TwoSubjectAnnotation
- getAlignDir()
  : broad.core.motif.PositionWeightMatrix
- getAlignedSequence()
  : broad.core.multiplealignment.MAFAlignment
  , broad.core.multiplealignment.MultipleAlignment
- getAlignedSequenceIds()
  : broad.core.multiplealignment.MAFAlignment
  , broad.core.multiplealignment.MultipleAlignment
- getAlignedSequences()
  : broad.core.multiplealignment.MAFAlignment
  , broad.core.multiplealignment.MultipleAlignment
- getAlignment()
  : broad.pda.seq.graph.Path
  , umms.core.alignment.PairedEndAlignmentFactory
  , umms.core.annotation.Gene
- getAlignmentChunkSize()
  : broad.core.siphy.ConservedPWMScanner
- getAlignmentDataModelStats()
  : broad.pda.seq.segmentation.ContinuousDataAlignmentModel
- getAlignmentEnd()
  : umms.core.alignment.AbstractPairedEndAlignment
  , umms.core.alignment.Alignment
  , umms.core.alignment.SingleEndAlignment
- getAlignmentIdxSizeAtCreation()
  : broad.pda.seq.alignment.StoredAlignmentStats
- getAlignmentSizeAtCreation()
  : broad.pda.seq.alignment.StoredAlignmentStats
- getAlignmentStart()
  : umms.core.alignment.AbstractPairedEndAlignment
  , umms.core.alignment.Alignment
  , umms.core.alignment.SingleEndAlignment
- getAlignmentType()
  : umms.core.model.JCSAlignmentModel
  , umms.core.readers.PairedEndReader
- getAllChromosomes()
  : broad.pda.feature.genome.DirectoryInstalledGenomeAssembly
- getAllDataValues()
  : broad.core.math.EmpiricalDistribution
- getAllIsoformExonSet()
  : broad.pda.gene.GeneWithIsoforms
- getAllIsoformIntronSet()
  : broad.pda.gene.GeneWithIsoforms
- getAllIsoforms()
  : broad.pda.annotation.Locus
  , broad.pda.gene.GeneWithIsoforms
- getAllMerged()
  : broad.pda.annotation.Locus
- getAllNonRandomChromosomes()
  : broad.pda.feature.genome.DirectoryInstalledGenomeAssembly
- getAllPairwiseAntisenseAlignments()
  : umms.core.alignment.FeatureSequenceAlignment
- getAllPairwiseSenseAlignments()
  : umms.core.alignment.FeatureSequenceAlignment
- getAllPaths()
  : broad.pda.seq.graph.ChromosomeWithBubblesJGraphT
- getAllPositions()
  : umms.core.annotation.Gene
- getAllPossibleAmplicons()
  : broad.core.primer3.isPCRLike
- getAllPossibleTargets()
  : umms.core.alignment.SmatchLike
- getAlphabetSize()
  : broad.core.motif.PositionWeightColumn
  , broad.core.siphy.EvolutionaryModel
- getAMap()
  : broad.core.math.Loess
- getAName()
  : broad.core.annotation.BasicTwoSubjectAnnotation
  , broad.core.annotation.TwoSubjectAnnotation
- getAnnotation()
  : broad.core.annotation.AnnotationReader< T extends GenomicAnnotation >
  , broad.pda.seq.clip.Pileup
  , umms.core.model.score.WindowScore.AbstractWindowScore
  , umms.core.model.score.WindowScore
- getAnnotationDirectory()
  : broad.pda.feature.genome.DirectoryInstalledGenomeAssembly
- getAnnotationList()
  : broad.core.annotation.AnnotationReader< T extends GenomicAnnotation >
  , broad.core.annotation.BasicGenomicAnnotationReader
- getAnnotationOverlappingRegion()
  : broad.pda.seq.segmentation.AlignmentDataModel
  , broad.pda.seq.segmentation.AlignmentDataModelStats
  , broad.pda.seq.segmentation.GenericAlignmentDataModel
- getAnnotationSetMap()
  : broad.pda.annotation.BEDFileParser
- getAnnotationsForSequence()
  : broad.core.annotation.AnnotationReader< T extends GenomicAnnotation >
- getAntisense()
  : broad.core.sequence.Sequence
- getArgString()
  : broad.core.parser.CommandLineParser
- getArray()
  : Jama.Matrix
- getArrayCopy()
  : Jama.Matrix
- getAsMatrix()
  : broad.core.multiplealignment.MultipleAlignment
- getAStart()
  : broad.core.annotation.BasicTwoSubjectAnnotation
  , broad.core.annotation.TwoSubjectAnnotation
- getAttribute()
  : umms.core.alignment.AbstractPairedEndAlignment
  , umms.core.alignment.Alignment
  , umms.core.alignment.SingleEndAlignment
  , umms.core.annotation.Gene
- getAttributes()
  : broad.core.annotation.GFF
  , umms.core.annotation.Gene
- getAutosomeGenomeSize()
  : broad.pda.feature.genome.DirectoryInstalledGenomeAssembly
- getAutosomeNumber()
  : broad.pda.feature.genome.DirectoryInstalledGenomeAssembly
- getAutosomes()
  : broad.pda.feature.genome.DirectoryInstalledGenomeAssembly
- getAverageCountPerPosition()
  : umms.core.model.AlignmentModel
  , umms.core.model.JCSAlignmentModel
- getAverageCountPerPositionWithThreshold()
  : umms.core.model.AlignmentModel
  , umms.core.model.JCSAlignmentModel
- getAverageCoverage()
  : umms.core.annotation.AbstractAnnotationCollection< T extends Annotation >
  , umms.core.annotation.AnnotationCollection< T extends Annotation >
  , umms.core.model.score.BasicScanStatisticScore
  , umms.core.model.score.MultiScore
  , umms.core.model.score.PoissonEnrichmentScore
  , umms.core.model.score.ScanStatisticScore
- getAverageScore()
  : broad.core.annotation.AnnotationReader< T extends GenomicAnnotation >
- getAvergeNumberOfReads()
  : broad.pda.seq.segmentation.GeneScore
- getB()
  : broad.core.annotation.BasicTwoSubjectAnnotation
  , broad.core.annotation.TwoSubjectAnnotation
- getBackgroundNucleotideFreqs()
  : broad.core.siphy.EvolutionaryModelParameters
- getBackwardEdges()
  : broad.pda.seq.graph.ChromosomeWithBubblesJGraphT
- getBackwardPaths()
  : broad.pda.seq.graph.ChromosomeWithBubblesJGraphT
- getBamFile()
  : umms.core.model.AlignmentModel
  , umms.core.model.JCSAlignmentModel
- getBaseCoverage()
  : broad.core.annotation.AnnotationReader< T extends GenomicAnnotation >
- getBaseIUB()
  : broad.core.motif.PositionWeightColumn
- getBasesCovered()
  : broad.core.annotation.AnnotationReader< T extends GenomicAnnotation >
  , broad.pda.seq.segmentation.AlignmentDataModel
  , broad.pda.seq.segmentation.ContinuousDataAlignmentModel
  , broad.pda.seq.segmentation.GenericAlignmentDataModel
  , umms.core.annotation.AbstractAnnotationCollection< T extends Annotation >
  , umms.core.annotation.AnnotationCollection< T extends Annotation >
  , umms.core.annotation.AnnotationList< T extends Annotation >
  , umms.core.model.AlignmentModel
  , umms.core.model.JCSAlignmentModel
- getBasesCoveredPerAlignment()
  : broad.pda.seq.segmentation.AlignmentDataModel
  , broad.pda.seq.segmentation.GenericAlignmentDataModel
- getBatch()
  : broad.pda.geneexpression.ExpressionExperimentInfo
- getBedScore()
  : umms.core.annotation.Gene
- getBEnd()
  : broad.core.annotation.BasicTwoSubjectAnnotation
  , broad.core.annotation.TwoSubjectAnnotation
- getBin()
  : broad.core.math.EmpiricalDistribution
- getBinEnd()
  : broad.core.math.EmpiricalDistribution
- getBinMidPoint()
  : broad.core.math.EmpiricalDistribution
- getBinNumber()
  : broad.core.math.EmpiricalDistribution
- getBinStart()
  : broad.core.math.EmpiricalDistribution
- getBlastHit()
  : broad.core.primer3.HairpinKmer
- getBlockIterator()
  : broad.core.multiplealignment.MAFAlignment
- getBlocks()
  : broad.core.annotation.BED
  , broad.core.annotation.GenomicAnnotation
  , broad.core.sequence.SequenceRegion
  , umms.core.annotation.Annotation
  , umms.core.annotation.BasicAnnotation
  , umms.core.annotation.CompoundInterval
- getBlockSizeStarts()
  : broad.core.annotation.BED
- getBName()
  : broad.core.annotation.BasicTwoSubjectAnnotation
  , broad.core.annotation.TwoSubjectAnnotation
- getBooleanArg()
  : broad.core.parser.CommandLineParser
- getBStart()
  : broad.core.annotation.BasicTwoSubjectAnnotation
  , broad.core.annotation.TwoSubjectAnnotation
- getCDNAs()
  : broad.pda.seq.segmentation.GenomeWithGaps
- getCDS()
  : umms.core.annotation.Gene
- getCDSEnd()
  : umms.core.annotation.Gene
- getCDSRegion()
  : umms.core.annotation.Gene
- getCDSStart()
  : umms.core.annotation.Gene
- getChildren()
  : umms.core.utils.AnnotationUtils
- getChr()
  : broad.core.sequence.SequenceRegion
  , umms.core.alignment.AbstractPairedEndAlignment
  , umms.core.alignment.Alignment
  , umms.core.alignment.SingleEndAlignment
  , umms.core.annotation.AbstractAnnotation
  , umms.core.annotation.Annotation
- getChrLength()
  : broad.pda.seq.segmentation.GenericAlignmentDataModel
- getChrLengths()
  : broad.pda.seq.segmentation.GenericAlignmentDataModel
- getChrNum()
  : umms.core.annotation.Gene
- getChromosome()
  : broad.core.annotation.BasicLightweightAnnotation
  , broad.core.annotation.LightweightGenomicAnnotation
  , broad.core.sequence.SequenceRegion
  , broad.pda.feature.genome.DirectoryInstalledGenomeAssembly
  , broad.pda.seq.graph.Path
  , umms.core.alignment.AbstractPairedEndAlignment
  , umms.core.alignment.SingleEndAlignment
- getChromosomeAnnotationMap()
  : broad.core.annotation.AnnotationReader< T extends GenomicAnnotation >
- getChromosomeBEDs()
  : broad.core.annotation.AnnotationReader< T extends GenomicAnnotation >
- getChromosomeIterator()
  : broad.core.annotation.AnnotationReader< T extends GenomicAnnotation >
  , broad.pda.annotation.BEDFileParser
- getChromosomeLength()
  : broad.pda.seq.graph.ChromosomeWithBubblesJGraphT
  , broad.pda.seq.segmentation.AlignmentDataModel
  , broad.pda.seq.segmentation.ContinuousDataAlignmentModel
  , broad.pda.seq.segmentation.GenericAlignmentDataModel
- getChromosomeLengths()
  : broad.pda.seq.segmentation.AlignmentDataModel
  , broad.pda.seq.segmentation.AlignmentDataModelStats
  , broad.pda.seq.segmentation.ContinuousDataAlignmentModel
  , broad.pda.seq.segmentation.GenericAlignmentDataModel
- getChromosomeNames()
  : umms.core.coordinatesystem.CoordinateSpace
  , umms.core.coordinatesystem.GenomicSpace
  , umms.core.coordinatesystem.MaskedGenomicSpace
  , umms.core.coordinatesystem.TranscriptInGenomicSpace
  , umms.core.coordinatesystem.TranscriptomeSpace
- getChromosomeNumber()
  : broad.pda.feature.genome.Chromosome
- getChromosomeReadIterator()
  : broad.pda.seq.segmentation.AlignmentDataModel
  , broad.pda.seq.segmentation.GenericAlignmentDataModel
- getChromosomes()
  : broad.pda.datastructures.LocationAwareMatrix
- getChromosomeString()
  : broad.core.annotation.BasicLightweightAnnotation
  , broad.core.annotation.LightweightGenomicAnnotation
  , broad.core.sequence.SequenceRegion
- getChromosomeTree()
  : broad.core.annotation.AnnotationReader< T extends GenomicAnnotation >
- getChromosomeTreeMap()
  : broad.core.annotation.AnnotationReader< T extends GenomicAnnotation >
- getChrReadIterator()
  : broad.pda.seq.segmentation.GenericAlignmentDataModel
- getChrSequence()
  : broad.core.sequence.SequenceUtils
- getChrSequencesFromFasta()
  : broad.core.sequence.FastaSequenceIO
- getChrSizesFile()
  : broad.core.sequence.SequenceUtils
- getChrTree()
  : broad.pda.annotation.BEDFileParser
- getChunkEnd()
  : broad.pda.seq.segmentation.AlignmentDataModel
  , broad.pda.seq.segmentation.AlignmentDataModelStats
  , broad.pda.seq.segmentation.GenericAlignmentDataModel
- getChunkStart()
  : broad.pda.seq.segmentation.AlignmentDataModel
  , broad.pda.seq.segmentation.AlignmentDataModelStats
  , broad.pda.seq.segmentation.GenericAlignmentDataModel
- getCigarString()
  : umms.core.alignment.SingleEndAlignment
- getClosest()
  : broad.pda.datastructures.LocationAwareMatrix
  , umms.core.annotation.AnnotationList< T extends Annotation >
- getClosestDownstream()
  : broad.pda.annotation.BEDFileParser
  , umms.core.annotation.AnnotationList< T extends Annotation >
- getClosestNonOverlapping()
  : umms.core.annotation.AnnotationList< T extends Annotation >
- getClosestUpstream()
  : broad.pda.annotation.BEDFileParser
  , umms.core.annotation.AnnotationList< T extends Annotation >
- getClusterInfo()
  : broad.pda.geneexpression.clustering.Kmeans
- getCol()
  : jaligner.Cell
- getColoumnName()
  : broad.core.datastructures.MatrixWithHeaders
- getColumn()
  : broad.core.datastructures.MatrixWithHeaders
  , broad.core.multiplealignment.MAFAlignment
  , broad.core.multiplealignment.MultipleAlignment
  , Jama.Matrix
- getColumnDimension()
  : Jama.Matrix
- getColumnNames()
  : broad.core.datastructures.MatrixWithHeaders
- getColumnNum()
  : broad.pda.geneexpression.agilent.AgilentProbe
- getColumnPackedCopy()
  : Jama.Matrix
- getColumnsAsVector()
  : broad.core.multiplealignment.MAFAlignment
  , broad.core.multiplealignment.MultipleAlignment
- getComaptibleBetweenAny2Sets()
  : broad.pda.annotation.Locus
- getComaptibleIsoforms()
  : broad.pda.annotation.Locus
- getCombinedPvalue()
  : broad.core.math.FisherCombinedProbabilityTest
- getComplement()
  : broad.core.motif.PositionWeightColumn
- getConcatenatedSubAlignment()
  : broad.core.multiplealignment.MultipleAlignment
- getConsensus()
  : broad.core.motif.PositionWeightMatrix
- getConsensusSequence()
  : broad.core.motif.SequenceMotif
- getConsistencyProblems()
  : broad.core.hmm.MarkovModel< T >
- getContainingSequenceId()
  : broad.core.sequence.SequenceRegion
- getControlClass()
  : broad.core.util.ParseGCTFile
- getCoordinateAtPosition()
  : umms.core.annotation.CompoundInterval
- getCoordinateSpace()
  : broad.pda.seq.protection.MultiSampleScanPeakCaller
  , broad.pda.seq.rap.GenomeCommandLineProgram
  , broad.pda.seq.rap.RAPPeakCaller
  , umms.core.annotation.AnnotationCollection< T extends Annotation >
  , umms.core.annotation.AnnotationList< T extends Annotation >
  , umms.core.model.AlignmentModel
  , umms.core.model.JCSAlignmentModel
  , umms.core.model.score.ScanStatisticScore
- getCoreMotifs()
  : broad.core.motif.SequenceMotif
- getCount()
  : broad.pda.seq.segmentation.ContinuousDataAlignmentModel
  , umms.core.annotation.AbstractAnnotationCollection< T extends Annotation >
  , umms.core.annotation.AnnotationCollection< T extends Annotation >
  , umms.core.annotation.AnnotationList< T extends Annotation >
  , umms.core.model.AlignmentModel.AlignmentCount
  , umms.core.model.AlignmentModel
  , umms.core.model.JCSAlignmentModel.AlignmentCount
  , umms.core.model.JCSAlignmentModel
  , umms.core.model.score.AbstractListScore
  , umms.core.model.score.CountScore
  , umms.core.model.score.EndInWindowCountScore
  , umms.core.model.score.StartInWindowCountScore
- getCountByBinStart()
  : broad.core.math.EmpiricalDistribution
- getCountExcludingRegion()
  : umms.core.annotation.AnnotationCollection< T extends Annotation >
  , umms.core.annotation.AnnotationList< T extends Annotation >
  , umms.core.model.AlignmentModel
  , umms.core.model.JCSAlignmentModel
- getCountProcessor()
  : umms.core.annotation.AbstractAnnotationCollection< T extends Annotation >
- getCounts()
  : broad.pda.seq.segmentation.AlignmentDataModel
  , broad.pda.seq.segmentation.AlignmentDataModelStats
  , broad.pda.seq.segmentation.ContinuousDataAlignmentModel
  , broad.pda.seq.segmentation.GenericAlignmentDataModel
- getCountScore()
  : broad.pda.datastructures.Alignments
  , umms.core.annotation.Gene
- getCountsOfUniqueOverlappers()
  : broad.pda.seq.segmentation.AlignmentDataModel
  , broad.pda.seq.segmentation.AlignmentDataModelStats
  , broad.pda.seq.segmentation.GenericAlignmentDataModel
- getCountsOnBase()
  : broad.pda.seq.segmentation.AlignmentDataModel
  , broad.pda.seq.segmentation.GenericAlignmentDataModel
- getCountsOnWindow()
  : broad.pda.seq.segmentation.GenericAlignmentDataModel
- getCountsPerAlignment()
  : broad.pda.seq.segmentation.AlignmentDataModel
  , broad.pda.seq.segmentation.AlignmentDataModelStats
  , broad.pda.seq.segmentation.GenericAlignmentDataModel
- getCountsPerAlignmentForPolyA()
  : broad.pda.seq.segmentation.GenericAlignmentDataModel
- getCountsPerAlignmentFullyContained()
  : broad.pda.seq.segmentation.AlignmentDataModel
  , broad.pda.seq.segmentation.GenericAlignmentDataModel
- getCountsPerAlignmentUncached()
  : broad.pda.seq.segmentation.GenericAlignmentDataModel
- getCountsPerAlignmentWithSameEndpointForPolyA()
  : broad.pda.seq.segmentation.GenericAlignmentDataModel
- getCountsPerAnnotationtranded()
  : broad.pda.seq.segmentation.AlignmentDataModel
  , broad.pda.seq.segmentation.GenericAlignmentDataModel
- getCountsPerBp()
  : broad.pda.seq.segmentation.ContinuousDataAlignmentModel
- getCountsPerPosition()
  : umms.core.model.AlignmentModel
  , umms.core.model.JCSAlignmentModel
- getCountsStrandedPerPosition()
  : umms.core.model.AlignmentModel
  , umms.core.model.JCSAlignmentModel
- getCountStrandedExcludingRegion()
  : umms.core.annotation.AnnotationCollection< T extends Annotation >
  , umms.core.annotation.AnnotationList< T extends Annotation >
  , umms.core.model.AlignmentModel
  , umms.core.model.JCSAlignmentModel
- getCountsWithinExons()
  : broad.pda.seq.segmentation.AlignmentDataModel
  , broad.pda.seq.segmentation.GenericAlignmentDataModel
- getCoverage()
  : broad.pda.seq.graph.Path
- getCtrlCoordSpace()
  : umms.core.model.score.MultiScore
- getCtrlCount()
  : umms.core.model.score.BinomialEnrichmentScore
  , umms.core.model.score.MultiScore
  , umms.core.model.score.PoissonEnrichmentScore
- getCtrlLambda()
  : umms.core.model.score.PoissonEnrichmentScore
- getCtrlRegionCount()
  : umms.core.model.score.BinomialEnrichmentScore
  , umms.core.model.score.MultiScore
- getCtrlRegionTotal()
  : umms.core.model.score.PoissonEnrichmentScore
- getCtrlSampleLambda()
  : umms.core.model.score.PoissonEnrichmentScore
- getCtrlTotal()
  : umms.core.model.score.PoissonEnrichmentScore
- getCumulativeProbability()
  : broad.core.math.EmpiricalDistribution
- getCurrentBase()
  : broad.core.sequence.AbstractFastaParser
- getCurrentQuality()
  : broad.core.sequence.FastaQualityParser
- getCurrentRelease()
  : jaligner.util.Commons
- getCurrentSequenceId()
  : broad.core.sequence.AbstractFastaParser
- getD()
  : Jama.EigenvalueDecomposition
- getData()
  : broad.core.datastructures.MatrixWithHeaders
  , broad.pda.seq.clip.SampleData
  , broad.pda.seq.protection.SampleData
  , broad.pda.seq.segmentation.AlignmentDataModelStats
  , broad.pda.seq.segmentation.ContinuousDataAlignmentModel
- getDataForAlignment()
  : broad.pda.seq.segmentation.ContinuousDataAlignmentModel
- getDataForGene()
  : broad.pda.seq.segmentation.ContinuousDataAlignmentModel
- getDataModel()
  : broad.core.sequence.SequenceUtils
- getDataType1()
  : broad.pda.enrichment.EnrichmentMetric
- getDataType2()
  : broad.pda.enrichment.EnrichmentMetric
- getDataTypes()
  : broad.pda.enrichment.EnrichmentMetric
- getDebugInfo()
  : umms.core.model.score.MultiScore
  , umms.core.model.score.ScanStatisticScore
- getDefaultFile()
  : umms.core.writers.PairedEndWriter
- getDenominatorCount()
  : umms.core.model.score.NewRatioScore
  , umms.core.model.score.RatioScore
  , umms.core.model.score.WindowAllScore
- getDenominatorLambda()
  : umms.core.model.score.WindowAllScore
- getDenominatorRegionTotal()
  : umms.core.model.score.NewRatioScore
  , umms.core.model.score.RatioScore
  , umms.core.model.score.WindowAllScore
- getDenominatorRPKM()
  : umms.core.model.score.NewRatioScore
  , umms.core.model.score.RatioScore
  , umms.core.model.score.WindowAllScore
- getDenominatorScanPVal()
  : broad.pda.seq.rap.SkellamScore
  , umms.core.model.score.WindowAllScore
- getDenominatorTotal()
  : umms.core.model.score.NewRatioScore
  , umms.core.model.score.RatioScore
  , umms.core.model.score.WindowAllScore
- getDensity()
  : broad.core.math.EmpiricalDistribution
- getDepletionBinomialPValue()
  : umms.core.model.score.NewRatioScore
  , umms.core.model.score.RatioScore
  , umms.core.model.score.WindowAllScore
- getDepletionBinomialScore()
  : umms.core.model.score.RatioScore
- getDescription()
  : broad.pda.seq.fastq.FastqSequence
  , jaligner.Sequence
- getDirection()
  : jaligner.TracebackNode
- getDistancesToClosest()
  : broad.core.annotation.AnnotationReader< T extends GenomicAnnotation >
- getDistanceTo()
  : broad.core.annotation.BasicLightweightAnnotation
  , broad.core.annotation.LightweightGenomicAnnotation
  , broad.core.sequence.SequenceRegion
  , umms.core.annotation.AbstractAnnotation
  , umms.core.annotation.Annotation
  , umms.core.annotation.SingleInterval
- getDistanceTo3primeNeighbor()
  : broad.pda.annotation.Locus
- getDistanceTo5primeNeighbor()
  : broad.pda.annotation.Locus
- getDistanceToClosest()
  : broad.core.annotation.AnnotationReader< T extends GenomicAnnotation >
  , umms.core.annotation.AnnotationList< T extends Annotation >
- getDistributionOfHistogramValues()
  : broad.core.math.EmpiricalDistribution
- getDoubleArg()
  : broad.core.parser.CommandLineParser
- getDoublePivot()
  : Jama.LUDecomposition
- getdoubleronicCounts()
  : broad.pda.seq.segmentation.GeneCounts
- getDuplicateReadFlag()
  : umms.core.alignment.SingleEndAlignment.WrapSamRecord
- getEdgeList()
  : broad.pda.seq.graph.Path
- getEdges()
  : broad.pda.seq.graph.ChromosomeWithBubblesJGraphT
  , broad.pda.seq.graph.Path
- getElements()
  : broad.core.siphy.PhastconsReader
- getEmissionLogProbability()
  : broad.core.hmm.MarkovState< T >
- getEmissionProbability()
  : broad.core.hmm.MarkovState< T >
- getEmpiricalMaxStatisticDistribution()
  : broad.core.math.EmpiricalDistribution
- getEmptyEmpiricalDistribution()
  : broad.pda.seq.rap.BuildRatioNullDistribution
- getEncodedSequence()
  : broad.core.sequence.Sequence
- getEnd()
  : broad.core.annotation.LightweightGenomicAnnotation
  , broad.core.datastructures.Interval
  , broad.core.sequence.Sequence
  , broad.core.sequence.SequenceRegion
  , broad.pda.seq.graph.ChromosomeWithBubblesJGraphT
  , broad.pda.seq.graph.Path
  , umms.core.alignment.AbstractPairedEndAlignment
  , umms.core.alignment.Alignment
  , umms.core.annotation.Annotation
  , umms.core.annotation.BasicAnnotation
  , umms.core.annotation.CompoundInterval
- getEndVertex()
  : broad.pda.seq.graph.Path
- getEnrichment()
  : broad.pda.seq.segmentation.GeneScore
  , umms.core.model.score.ScanStatisticScore
- getEnrichmentBinomialPValue()
  : umms.core.model.score.NewRatioScore
  , umms.core.model.score.RatioScore
  , umms.core.model.score.WindowAllScore
- getEnrichmentBinomialScore()
  : umms.core.model.score.RatioScore
- getEnrichmentOverControl()
  : umms.core.model.score.BinomialEnrichmentScore
  , umms.core.model.score.MultiScore
- getEnrichmentOverGene()
  : broad.pda.seq.clip.SampleData
  , broad.pda.seq.protection.SampleData
- getEntireChromosome()
  : umms.core.coordinatesystem.CoordinateSpace
  , umms.core.coordinatesystem.GenomicSpace
  , umms.core.coordinatesystem.MaskedGenomicSpace
  , umms.core.coordinatesystem.TranscriptInGenomicSpace
  , umms.core.coordinatesystem.TranscriptomeSpace
- getEstimatedLibrarySize()
  : broad.pda.countreads.FastqLibraryStats
- getEstimationWindow()
  : broad.core.siphy.TreeScalerIO
- getEval()
  : broad.core.motif.meme.MemeMotif
- getExactIsoform()
  : broad.pda.annotation.BEDFileParser
- getExonAnnotationOverlappingRegion()
  : broad.pda.seq.segmentation.AlignmentDataModel
  , broad.pda.seq.segmentation.AlignmentDataModelStats
  , broad.pda.seq.segmentation.GenericAlignmentDataModel
- getExonicCounts()
  : broad.pda.seq.segmentation.GeneCounts
- getExons()
  : umms.core.annotation.Gene
- getExonSet()
  : umms.core.annotation.Gene
- getExonsScores()
  : umms.core.annotation.Gene
- getExonTree()
  : umms.core.annotation.Gene
- getExonUnion()
  : broad.pda.annotation.Locus
- getExperimentBarcode()
  : broad.pda.geneexpression.ExpressionExperimentInfo
- getExperimentHybDate()
  : broad.pda.geneexpression.ExpressionExperimentInfo
- getExperimentName()
  : broad.pda.geneexpression.ExpressionExperimentInfo
- getExperimentPlatePosition()
  : broad.pda.geneexpression.ExpressionExperimentInfo
- getExpressionScanPvalueCutoff()
  : broad.pda.seq.clip.SampleData
  , broad.pda.seq.protection.SampleData
- getExtend()
  : jaligner.Alignment
- getExtraFields()
  : umms.core.annotation.Gene
- getExtraScore()
  : broad.core.annotation.BasicLightweightAnnotation
  , broad.core.annotation.LightweightGenomicAnnotation
  , broad.core.sequence.SequenceRegion
- getExtraScores()
  : broad.core.annotation.BasicLightweightAnnotation
  , broad.core.annotation.LightweightGenomicAnnotation
  , broad.core.sequence.SequenceRegion
- getFactory()
  : broad.core.annotation.AnnotationFactoryFactory
- getFDR()
  : broad.core.math.FDRDistribution
  , broad.pda.differentialExpression.RunDifferentialExpression
- getFDRDistribution()
  : broad.pda.differentialExpression.DifferentialExpression
- getFDRMatrix()
  : broad.pda.differentialExpression.DifferentialExpression
- getFeature()
  : broad.core.annotation.GFF
- getFieldCount()
  : broad.core.parser.StringParser
- getFileSeparator()
  : jaligner.util.Commons
- getFileSize()
  : broad.core.sequence.AbstractFastaParser
- getFinalMValues()
  : broad.core.math.Loess
- getFirstExon()
  : umms.core.annotation.Gene
- getFirstFragmentPositionStranded()
  : umms.core.alignment.AbstractPairedEndAlignment
  , umms.core.alignment.Alignment
  , umms.core.alignment.SingleEndAlignment
- getFirstGene()
  : umms.core.alignment.FeatureSequenceAlignment.UnorderedGenePair
- getFirstIntersectLeftFromMedian()
  : broad.core.math.EmpiricalDistribution
- getFirstIntersectRightFromMedian()
  : broad.core.math.EmpiricalDistribution
- getFirstIntron()
  : umms.core.annotation.Gene
- getFirstMate()
  : umms.core.alignment.AbstractPairedEndAlignment
- getFirstNBPs()
  : broad.pda.seq.fastq.FastqSequence
- getFirstSequence()
  : broad.pda.rnai.ExtractSequence
- getFirstValue()
  : broad.core.annotation.GFF
- getFits()
  : broad.core.siphy.StationaryDistributionIO
- getFivePrimeBases()
  : broad.core.annotation.BasicGenomicAnnotation
  , broad.core.annotation.GenomicAnnotation
  , broad.core.sequence.SequenceRegion
- getFlagsAndValues()
  : broad.core.parser.CommandLineParser
- getFlankingBlocks()
  : umms.core.annotation.BasicAnnotation
- getFloatArg()
  : broad.core.parser.CommandLineParser
- getFoldMatrix()
  : broad.pda.differentialExpression.DifferentialExpression
- getFormat()
  : jaligner.formats.FormatFactory
- getFormats()
  : jaligner.formats.FormatFactory
- getForwardEdges()
  : broad.pda.seq.graph.ChromosomeWithBubblesJGraphT
- getForwardTargetRegions()
  : umms.core.alignment.SmatchLike
- getForwardTargets()
  : umms.core.alignment.SmatchLike
- getFragment()
  : umms.core.alignment.AbstractPairedEndAlignment
  , umms.core.alignment.Alignment
  , umms.core.alignment.SingleEndAlignment
  , umms.core.coordinatesystem.CoordinateSpace
  , umms.core.coordinatesystem.GenomicSpace
  , umms.core.coordinatesystem.MaskedGenomicSpace
  , umms.core.coordinatesystem.TranscriptInGenomicSpace
  , umms.core.coordinatesystem.TranscriptomeSpace
- getFragmentEnd()
  : umms.core.alignment.AbstractPairedEndAlignment
  , umms.core.alignment.Alignment
  , umms.core.alignment.SingleEndAlignment
- getFragmentEndsFromBamFile()
  : broad.pda.seq.clip.Pileup
- getFragmentLengthFilterData()
  : broad.pda.seq.clip.SampleData
  , broad.pda.seq.protection.SampleData
- getFragmentLengthGeneCount()
  : broad.pda.seq.clip.SampleData
- getFragmentMidpoint()
  : umms.core.alignment.AbstractPairedEndAlignment
  , umms.core.alignment.Alignment
  , umms.core.alignment.SingleEndAlignment
- getFragmentSize()
  : umms.core.alignment.AbstractPairedEndAlignment
  , umms.core.alignment.Alignment
  , umms.core.alignment.SingleEndAlignment
- getFragmentStart()
  : umms.core.alignment.AbstractPairedEndAlignment
  , umms.core.alignment.Alignment
  , umms.core.alignment.SingleEndAlignment
- getFragmentStrand()
  : umms.core.alignment.AbstractPairedEndAlignment
  , umms.core.alignment.Alignment
  , umms.core.alignment.SingleEndAlignment
- getFrame()
  : broad.core.annotation.GFF
- getFullInfoString()
  : broad.core.sequence.SequenceRegion
  , umms.core.annotation.AbstractAnnotation
  , umms.core.annotation.Annotation
- getFullIntervalTreeAsAnnotation()
  : broad.pda.seq.segmentation.AlignmentDataModel
  , broad.pda.seq.segmentation.GenericAlignmentDataModel
- getFullPrintableAlignment()
  : umms.core.alignment.SmithWatermanAlignment
- getFullyContainedNumberOfReads()
  : broad.pda.seq.segmentation.GeneScore
  , umms.core.model.score.BasicScanStatisticScore
  , umms.core.model.score.ScanStatisticScore
- getFWER()
  : broad.pda.differentialExpression.FWERDistribution
- getFWERMatrix()
  : broad.pda.differentialExpression.DifferentialExpression
- getGappedSeqsInWindowMatrix()
  : broad.core.siphy.ConservationUtils
- getGappedSize()
  : umms.core.annotation.Gene
- getGaps()
  : broad.pda.seq.segmentation.GenomeWithGaps
  , jaligner.Alignment
- getGaps1()
  : jaligner.Alignment
- getGaps2()
  : jaligner.Alignment
- getGapsSize()
  : broad.core.sequence.Sequence
- getGene()
  : broad.pda.annotation.BEDFileParser
  , umms.core.model.score.MultiScore
- getGeneAverageCoverage()
  : broad.pda.seq.clip.SampleData
  , broad.pda.seq.protection.SampleData
- getGeneByName()
  : broad.pda.annotation.BEDFileParser
- getGeneCount()
  : broad.pda.seq.clip.SampleData
  , broad.pda.seq.protection.SampleData
- getGeneCounts()
  : broad.pda.seq.segmentation.AlignmentDataModel
  , broad.pda.seq.segmentation.AlignmentDataModelStats
  , broad.pda.seq.segmentation.ContinuousDataAlignmentModel
  , broad.pda.seq.segmentation.GenericAlignmentDataModel
- getGeneCountsWithoutDatasetTotals()
  : broad.pda.seq.segmentation.AlignmentDataModelStats
  , broad.pda.seq.segmentation.ContinuousDataAlignmentModel
- getGeneDescription()
  : broad.pda.geneexpression.agilent.AgilentProbe
- getGeneExpression()
  : broad.pda.seq.segmentation.AlignmentDataModelStats
  , broad.pda.seq.segmentation.ContinuousDataAlignmentModel
- getGeneIDIsoMap()
  : broad.pda.annotation.GTFFileParser
- getGeneLength()
  : broad.pda.seq.segmentation.GeneScore
- getGeneName()
  : broad.pda.geneexpression.agilent.AgilentProbe
  , broad.pda.rnai.RNAiGeneAnnotation
- getGenePaths()
  : broad.pda.seq.graph.ChromosomeWithBubblesJGraphT
- getGenePval()
  : broad.pda.seq.clip.GenomeSpaceSampleData
- getGeneRandNeighbors()
  : broad.pda.gene.NeighborsNullModel
- getGenes()
  : broad.core.sequence.TranscribedRegions
- GetGenes()
  : broad.pda.annotation.BEDFileParser
- getGenes()
  : broad.pda.seq.rap.GenomeCommandLineProgram
- getGeneScanPval()
  : broad.pda.seq.clip.SampleData
  , broad.pda.seq.protection.GenomeSpaceSampleData
  , broad.pda.seq.protection.SampleData
- getGenesPassingFDR()
  : broad.pda.differentialExpression.DifferentialExpression
  , broad.pda.differentialExpression.RunDifferentialExpression
- GetGenesWithIsoforms()
  : broad.pda.annotation.BEDFileParser
- getGenomeSequence()
  : broad.pda.rnai.ExtractSequence
- getGenomeSize()
  : broad.pda.feature.genome.DirectoryInstalledGenomeAssembly
- getGenomicLength()
  : umms.core.annotation.Gene
- getGenomicRegionOverlappers()
  : broad.pda.annotation.BEDFileParser
- getGenomicSpace()
  : broad.pda.seq.rap.rna.TranscriptomeCommandLineProgram
- getGlobalCount()
  : umms.core.annotation.AnnotationCollection< T extends Annotation >
  , umms.core.annotation.AnnotationList< T extends Annotation >
  , umms.core.model.AlignmentModel
  , umms.core.model.JCSAlignmentModel
- getGlobalCountReferenceSeqs()
  : umms.core.model.AlignmentModel
  , umms.core.model.JCSAlignmentModel
- getGlobalEnrichment()
  : umms.core.model.score.BasicScanStatisticScore
- getGlobalLambda()
  : umms.core.model.AlignmentModel
  , umms.core.model.JCSAlignmentModel
  , umms.core.model.score.BasicScanStatisticScore
  , umms.core.model.score.MultiScore
  , umms.core.model.score.ScanStatisticScore
- getGlobalLength()
  : umms.core.model.AlignmentModel
  , umms.core.model.JCSAlignmentModel
  , umms.core.model.score.BasicScanStatisticScore
  , umms.core.model.score.MultiScore
  , umms.core.model.score.PoissonEnrichmentScore
  , umms.core.model.score.ScanStatisticScore
- getGlobalNumReads()
  : umms.core.model.AlignmentModel
  , umms.core.model.JCSAlignmentModel
- getGlobalNumReadsReferenceSeqs()
  : umms.core.model.AlignmentModel
  , umms.core.model.JCSAlignmentModel
- getGlobalPairedFragments()
  : umms.core.model.AlignmentModel
  , umms.core.model.JCSAlignmentModel
- getGlobalRPKMConstant()
  : broad.pda.seq.alignment.StoredAlignmentStats
  , broad.pda.seq.segmentation.AlignmentDataModelStats
- getGraph()
  : broad.pda.seq.graph.Path
- getGroup()
  : broad.core.annotation.GFF
- getGroupIndexes()
  : broad.core.util.ParseGCTFile
- getGTFAttr()
  : broad.core.annotation.GFF
- getGTFGeneTranscriptMap()
  : broad.pda.annotation.GTFFileParser
- getH()
  : Jama.QRDecomposition
- getHeader()
  : broad.core.multiplealignment.MAFAlignment
  , broad.pda.samtools.SAMPairedEndFileReader
  , umms.core.alignment.AbstractPairedEndAlignment
  , umms.core.alignment.Alignment
  , umms.core.alignment.SingleEndAlignment
  , umms.core.model.JCSAlignmentModel
  , umms.core.readers.PairedEndReader
- getHistogram()
  : broad.core.math.EmpiricalDistribution
- getId()
  : broad.core.annotation.BasicGenomicAnnotation
  , broad.core.annotation.GenomicAnnotation
  , broad.core.motif.SequenceMotif
  , broad.core.sequence.Sequence
  , broad.core.sequence.SequenceRegion
  , broad.pda.enrichment.EnrichmentMetric
  , jaligner.formats.Format
  , jaligner.matrix.Matrix
  , jaligner.Sequence
- getIdentity()
  : jaligner.Alignment
- getImagEigenvalues()
  : Jama.EigenvalueDecomposition
- getIncidentEdges()
  : broad.pda.seq.graph.ChromosomeWithBubblesJGraphT
- getIndecesForRowDescription()
  : broad.core.datastructures.MatrixWithHeaders
- getIndex()
  : broad.core.datastructures.IntervalTree< V >
  , broad.core.multiplealignment.MAFAlignment
- getIndexFor()
  : broad.core.parser.StringParser
- getIndexGroup()
  : broad.core.util.ParseGCTFile
- getInformationContent()
  : broad.core.motif.PositionWeightColumn
- getInitialStateTransitionProbabilities()
  : broad.core.hmm.MarkovModel< T >
- getInOrUnorientedEdges()
  : broad.pda.seq.graph.ChromosomeWithBubblesJGraphT
- getInstance()
  : jaligner.formats.FormatFactory
- getIntArg()
  : broad.core.parser.CommandLineParser
- getIntervalBetweenReads()
  : umms.core.alignment.AbstractPairedEndAlignment
  , umms.core.alignment.Alignment
  , umms.core.alignment.SingleEndAlignment
- getIntervalTree()
  : broad.pda.seq.segmentation.AlignmentDataModel
  , broad.pda.seq.segmentation.AlignmentDataModelStats
  , broad.pda.seq.segmentation.GenericAlignmentDataModel
- getIntervalTreeCached()
  : broad.pda.seq.segmentation.AlignmentDataModel
  , broad.pda.seq.segmentation.AlignmentDataModelStats
  , broad.pda.seq.segmentation.GenericAlignmentDataModel
- getIntervalTreeTruncated()
  : broad.pda.seq.segmentation.GenericAlignmentDataModel
- getIntervalTreeTruncatedCached()
  : broad.pda.seq.segmentation.AlignmentDataModel
  , broad.pda.seq.segmentation.AlignmentDataModelStats
  , broad.pda.seq.segmentation.GenericAlignmentDataModel
- getIntervalTreeWithIsoforoms()
  : broad.pda.annotation.BEDFileParser
- getIntronCounts()
  : umms.core.model.AlignmentModel
  , umms.core.model.JCSAlignmentModel
- getIntrons()
  : umms.core.annotation.Gene
- getIntronsBlocks()
  : umms.core.annotation.Gene
- getIntronSet()
  : umms.core.annotation.Gene
- getIntronTranscript()
  : umms.core.annotation.Gene
- getIOHelper()
  : broad.core.multiplealignment.MultipleAlignment
- getIsComaptibleBetweenAny2Sets()
  : broad.pda.annotation.Locus
- getIsComaptibleBetweenSubsets()
  : broad.pda.annotation.Locus
- getIsComaptibleWithRefPerSet()
  : broad.pda.annotation.Locus
- getIsFirstMate()
  : umms.core.alignment.SingleEndAlignment
- getIsoformMap()
  : umms.core.scripture.BuildScriptureCoordinateSpace
- getIsoforms()
  : umms.core.annotation.Gene
- getIsPartiallyComaptibleWithRefPerSet()
  : broad.pda.annotation.Locus
- getJAlignerInfo()
  : jaligner.util.Commons
- getKmerEndPosition()
  : broad.core.primer3.HairpinKmer
- getKmerSequence()
  : broad.core.primer3.HairpinKmer
- getKmerStartPosition()
  : broad.core.primer3.HairpinKmer
- getKSEnrichments()
  : broad.projection.gsa.GeneSetEnrichment
- getKSFDR()
  : broad.projection.gsa.GeneSetEnrichment
- getKSScores()
  : broad.projection.gsa.GSAScoring
- getKSStatistic()
  : broad.core.math.EmpiricalDistribution
  , broad.core.math.Statistics
- getL()
  : Jama.CholeskyDecomposition
  , Jama.LUDecomposition
- getLambda()
  : broad.pda.seq.alignment.StoredAlignmentStats
  , broad.pda.seq.graph.ChromosomeWithBubblesJGraphT
  , broad.pda.seq.segmentation.AlignmentDataModelStats
  , broad.pda.seq.segmentation.ContinuousDataAlignmentModel
- getLargestParent()
  : umms.core.utils.AnnotationUtils
- getLastExon()
  : umms.core.annotation.Gene
- getLastExons()
  : broad.core.annotation.AnnotationReader< T extends GenomicAnnotation >
- getLastFragmentPositionStranded()
  : umms.core.alignment.AbstractPairedEndAlignment
  , umms.core.alignment.Alignment
  , umms.core.alignment.SingleEndAlignment
- getLastIntron()
  : umms.core.annotation.Gene
- getLastModifiedAlignmentIdxTimeStampAtCreation()
  : broad.pda.seq.alignment.StoredAlignmentStats
- getLastModifiedAlignmentTimeStampAtCreation()
  : broad.pda.seq.alignment.StoredAlignmentStats
- getLastModifiedStoredTimeStamp()
  : broad.pda.seq.alignment.StoredAlignmentStats
- getLeaves()
  : broad.core.datastructures.GenericRootedTree< T >
- getLeft()
  : broad.pda.geneexpression.clustering.Cluster
- getLeftHighInfoStart()
  : broad.core.motif.PositionWeightMatrix
- getLeftNeighbor()
  : broad.pda.gene.NeighborAnalysis
- getLength()
  : broad.core.annotation.BasicGenomicAnnotation
  , broad.core.annotation.GenomicAnnotation
  , broad.core.datastructures.Interval
  , broad.core.sequence.Sequence
  , broad.core.sequence.SequenceRegion
  , broad.pda.seq.fastq.FastqSequence
  , jaligner.Alignment
  , jaligner.TracebackNode
  , umms.core.coordinatesystem.CoordinateSpace
  , umms.core.coordinatesystem.GenomicSpace
  , umms.core.coordinatesystem.MaskedGenomicSpace
  , umms.core.coordinatesystem.TranscriptInGenomicSpace
  , umms.core.coordinatesystem.TranscriptomeSpace.GeneTree
  , umms.core.coordinatesystem.TranscriptomeSpace
- getLengthOnReference()
  : broad.core.sequence.SequenceRegion
  , umms.core.annotation.AbstractAnnotation
  , umms.core.annotation.Annotation
- getLineSeparator()
  : jaligner.util.Commons
- getLlr()
  : broad.core.motif.meme.MemeMotif
- getLocalLambda()
  : broad.pda.seq.graph.Path
  , broad.pda.seq.segmentation.GeneScore
  , umms.core.model.score.BasicScanStatisticScore
  , umms.core.model.score.ScanStatisticScore
- getLocalRate()
  : broad.pda.seq.graph.ChromosomeWithBubblesJGraphT
- getLocalRpkmConstant()
  : broad.pda.seq.alignment.StoredAlignmentStats
- getLocalSampleLambda()
  : umms.core.model.score.PoissonEnrichmentScore
- getLocationString()
  : broad.core.annotation.BasicLightweightAnnotation
  , broad.core.annotation.GenomicAnnotation
  , broad.core.sequence.SequenceRegion
- getLog2Ratio()
  : umms.core.model.score.NewRatioScore
  , umms.core.model.score.RatioScore
  , umms.core.model.score.WindowAllScore
- getLog2RegionRatio()
  : umms.core.model.score.NewRatioScore
  , umms.core.model.score.RatioScore
  , umms.core.model.score.WindowAllScore
- getLogger()
  : broad.pda.seq.clip.SampleData
  , broad.pda.seq.protection.SampleData
- getLogLikelihood()
  : broad.core.motif.PositionWeightMatrix
  , broad.core.siphy.TreeScaler
- getLogLikelihoodRatio()
  : broad.core.siphy.Fit
- getLogProb()
  : broad.core.motif.PositionWeightColumn
- getLogProcessedSignal()
  : broad.pda.geneexpression.agilent.AgilentProbe
- getLogRatio()
  : broad.core.motif.PositionWeightColumn
- getLogRatioWeightedByNegEntropy()
  : broad.core.motif.PositionWeightColumn
- getLongestIntornChainIso()
  : broad.pda.annotation.Locus
- getLongestPerfectMatch()
  : jaligner.Alignment
- getLongestUngappedAlignment()
  : jaligner.Alignment
- getMap()
  : broad.core.math.Loess
- getMappingQuality()
  : umms.core.alignment.AbstractPairedEndAlignment
  , umms.core.alignment.Alignment
  , umms.core.alignment.SingleEndAlignment
  , umms.core.alignment.SingleEndAlignment.WrapSamRecord
- getMarkupLine()
  : jaligner.Alignment
- getMaskedRegions()
  : broad.pda.seq.alignment.StoredAlignmentStats
- getMaskedRegionsSize()
  : broad.pda.seq.segmentation.AlignmentDataModelStats
- getMatch()
  : umms.core.annotation.PSLRecord
- getMatches()
  : broad.core.motif.meme.MemeMotif
  , broad.core.sequence.Sequence
- getMatrices()
  : broad.core.motif.PositionWeightMatrixIO
- getMatrix()
  : broad.pda.geneexpression.clustering.HierarchicalClustering
  , jaligner.Alignment
  , Jama.Matrix
- getMax()
  : broad.core.math.EmpiricalDistribution
- getMaxExonsCovered()
  : broad.pda.annotation.Locus
- getMaxMeanEnrichments()
  : broad.projection.gsa.GeneSetEnrichment
- getMaxMeanFDR()
  : broad.projection.gsa.GeneSetEnrichment
- getMaxMeanScores()
  : broad.projection.gsa.GSAScoring
- getMaxPctCovered()
  : broad.pda.annotation.Locus
- getMaxPctGenomeCovered()
  : broad.pda.annotation.Locus
- getMaxScoreAcrossAllIso()
  : broad.pda.annotation.Locus
- getMaxScorePerSet()
  : broad.pda.annotation.Locus
- getMean()
  : broad.core.math.EmpiricalDistribution
  , umms.core.model.score.AbstractListScore
- getMeanOverAllRows()
  : broad.core.datastructures.MatrixWithHeaders
- getMedian()
  : umms.core.model.score.AbstractListScore
- getMedianOfAllDataValues()
  : broad.core.math.EmpiricalDistribution
- getMedianOverAllRows()
  : broad.core.datastructures.MatrixWithHeaders
- getMedianReadSize()
  : umms.core.model.AlignmentModel
  , umms.core.model.JCSAlignmentModel
- getMembers()
  : broad.pda.geneexpression.clustering.Cluster
- getMerged()
  : broad.pda.annotation.Locus
  , broad.pda.gene.GeneWithIsoforms
- getMergedAnnotationMap()
  : broad.pda.annotation.BEDFileParser
- getMergedCopy()
  : broad.pda.annotation.BEDFileParser
- getMessage()
  : broad.core.hmm.BadModelException
- getMethod()
  : broad.pda.enrichment.EnrichmentMetric
- getMiddle()
  : broad.core.annotation.BasicLightweightAnnotation
  , broad.core.annotation.LightweightGenomicAnnotation
  , broad.core.sequence.SequenceRegion
- getMidpoint()
  : broad.core.sequence.SequenceRegion
- getMidPoint()
  : broad.pda.datastructures.Alignments
- getMidpoint()
  : umms.core.annotation.AbstractAnnotation
  , umms.core.annotation.Annotation
  , umms.core.annotation.BasicAnnotation
  , umms.core.utils.AnnotationUtils
- getMidpointGenomicCoords()
  : umms.core.annotation.Gene
- getMin()
  : broad.core.math.EmpiricalDistribution
- getMinimumMappingQuality()
  : broad.pda.seq.segmentation.AlignmentDataModel
  , broad.pda.seq.segmentation.AlignmentDataModelStats
  , broad.pda.seq.segmentation.GenericAlignmentDataModel
- getMinimumTreeLength()
  : broad.core.siphy.TreeScaler
- getMirScore()
  : broad.core.primer3.HairpinKmer
- getModel()
  : broad.core.siphy.TreeScaler
- getModelFilePath()
  : broad.pda.seq.segmentation.AlignmentDataModel
  , broad.pda.seq.segmentation.GenericAlignmentDataModel
- getModelParameters()
  : broad.core.siphy.EvolutionaryModel
- getMotif()
  : broad.core.motif.SequenceMotif
- getMotifNames()
  : broad.core.motif.misc.ComputeConservedMotifs
- getMotifs()
  : broad.core.motif.meme.MemeResultReader
- getName()
  : broad.core.annotation.Feature
  , broad.core.annotation.LightweightGenomicAnnotation
  , broad.core.datastructures.GenericRootedTree< T >
  , broad.core.hmm.MarkovState< T >
  , broad.core.motif.meme.MemeMotif
  , broad.core.motif.PositionWeightMatrix
  , broad.core.primer3.RestrictionEnzyme
  , broad.core.sequence.SequenceRegion
  , broad.pda.feature.genome.Chromosome
  , broad.pda.geneexpression.clustering.AbsolutePearsonFunction
  , broad.pda.geneexpression.clustering.ClusterDistanceFunction
  , broad.pda.geneexpression.clustering.EuclideanDistance
  , broad.pda.geneexpression.clustering.PearsonDistance
  , broad.pda.seq.fastq.FastqSequence
  , broad.pda.seq.graph.ChromosomeWithBubblesJGraphT
  , umms.core.annotation.Annotation
  , umms.core.annotation.BasicAnnotation
- getName1()
  : jaligner.Alignment
- getName2()
  : jaligner.Alignment
- getNameGeneMap()
  : broad.pda.annotation.BEDFileParser
- getNanostringProbeClasses()
  : broad.core.datastructures.MatrixWithHeaders
- getNanostringReplicateMap()
  : broad.core.datastructures.MatrixWithHeaders
- getNeighborDistance()
  : broad.pda.gene.NeighborAnalysis
- getNeighbors()
  : broad.pda.gene.NeighborAnalysis
- getNext()
  : broad.core.math.CombinationGenerator
- getNextClosest()
  : broad.pda.datastructures.LocationAwareMatrix
- getNextKmer()
  : broad.core.motif.PositionWeightMatrix
- getNextRandom()
  : broad.core.math.CombinationGenerator
- getNextWindow()
  : broad.pda.seq.segmentation.GenomeWithGaps
- getNodeCount()
  : broad.pda.seq.graph.Path
- getNodeElements()
  : broad.core.datastructures.GenericRootedTree< T >
- getNodeLikelihoodParameterMap()
  : broad.core.siphy.EvolutionaryModel
- getNodePair()
  : broad.pda.seq.graph.ChromosomeWithBubblesJGraphT
  , umms.core.scripture.OrientedChromosomeTranscriptGraph
- getNodes()
  : broad.core.datastructures.GenericRootedTree< T >
- getNominalPValue()
  : broad.pda.seq.segmentation.GeneScore
- getNominalPValueMatrix()
  : broad.pda.differentialExpression.DifferentialExpression
- getNonControl1PercentileSignal()
  : broad.pda.geneexpression.agilent.AgilentArrayStats
- getNonControl50PercentileSignal()
  : broad.pda.geneexpression.agilent.AgilentArrayStats
- getNonControl99PercentileSignal()
  : broad.pda.geneexpression.agilent.AgilentArrayStats
- getNonRandomTotalSize()
  : broad.pda.feature.genome.DirectoryInstalledGenomeAssembly
- getNormalizationFactor()
  : broad.pda.geneexpression.agilent.AgilentArrayStats
- getNormalizedKSEnrichments()
  : broad.projection.gsa.GeneSetEnrichment
- getNormalizedMaxMeanEnrichments()
  : broad.projection.gsa.GeneSetEnrichment
- getNormalizedScore()
  : umms.core.annotation.Gene
- getNormalizedSignal()
  : broad.pda.geneexpression.agilent.AgilentProbe
- getNullDistDegreesOfFreedom()
  : broad.core.math.FisherCombinedProbabilityTest
- getNum3PrimeComplementarity()
  : broad.core.primer3.Primer3PickingSummary
- getNum3PrimerStability()
  : broad.core.primer3.Primer3PickingSummary
- getNumberColumns()
  : broad.core.datastructures.MatrixWithHeaders
- getNumberMarkers()
  : broad.pda.seq.segmentation.AlignmentDataModelStats
  , broad.pda.seq.segmentation.ContinuousDataAlignmentModel
- getNumberOfAligningSequences()
  : broad.core.multiplealignment.MultipleAlignment
- getNumberOfBackgroundNonUniformFeaturesOverlimit()
  : broad.pda.geneexpression.agilent.AgilentArrayStats
- getNumberOfBackgroundPopulationOverlimit()
  : broad.pda.geneexpression.agilent.AgilentArrayStats
- getNumberOfBPs()
  : broad.pda.seq.segmentation.ContinuousDataAlignmentModel
- getNumberOfEdges()
  : broad.pda.seq.graph.Path
- getNumberOfFoundFeatures()
  : broad.pda.geneexpression.agilent.AgilentArrayStats
- getNumberOfIsoforms()
  : broad.pda.annotation.BEDFileParser
  , broad.pda.annotation.Locus
  , broad.pda.gene.GeneWithIsoforms
- getNumberOfMarkers()
  : broad.pda.seq.graph.ChromosomeWithBubblesJGraphT
- getNumberOfMatches()
  : jaligner.Alignment
- getNumberOfMatches2()
  : jaligner.Alignment
- getNumberOfNonUniformFeaturesOverlimit()
  : broad.pda.geneexpression.agilent.AgilentArrayStats
- getNumberOfPopulationOverlimit()
  : broad.pda.geneexpression.agilent.AgilentArrayStats
- getNumberOfReads()
  : broad.pda.seq.alignment.StoredAlignmentStats
  , broad.pda.seq.graph.ChromosomeWithBubblesJGraphT
  , broad.pda.seq.segmentation.AlignmentDataModelStats
  , broad.pda.seq.segmentation.ContinuousDataAlignmentModel
  , broad.pda.seq.segmentation.GeneScore
- getNumberOfReadsByChr()
  : broad.pda.seq.segmentation.AlignmentDataModelStats
- getNumberOfSaturatedProbes()
  : broad.pda.geneexpression.agilent.AgilentArrayStats
- getNumberOfSequences()
  : broad.pda.seq.fastq.FastqParser
- getNumberOfSplicedReads()
  : broad.pda.seq.segmentation.AlignmentDataModel
  , broad.pda.seq.segmentation.GenericAlignmentDataModel
- getNumberOfValuesLargerThan()
  : broad.core.math.EmpiricalDistribution
- getNumberPositions()
  : umms.core.annotation.Gene
- getNumberRows()
  : broad.core.datastructures.MatrixWithHeaders
- getNumCol()
  : broad.core.motif.PositionWeightMatrix
- getNumColdMeltingTemp()
  : broad.core.primer3.Primer3PickingSummary
- getNumColumns()
  : Jama.Matrix
- getNumEndPosToErase()
  : broad.core.motif.PositionWeightMatrix
- getNumeratorCount()
  : umms.core.model.score.NewRatioScore
  , umms.core.model.score.RatioScore
  , umms.core.model.score.WindowAllScore
- getNumeratorLambda()
  : umms.core.model.score.WindowAllScore
- getNumeratorRegionTotal()
  : umms.core.model.score.NewRatioScore
  , umms.core.model.score.RatioScore
  , umms.core.model.score.WindowAllScore
- getNumeratorRPKM()
  : umms.core.model.score.NewRatioScore
  , umms.core.model.score.RatioScore
  , umms.core.model.score.WindowAllScore
- getNumeratorScanPVal()
  : broad.pda.seq.rap.SkellamScore
  , umms.core.model.score.WindowAllScore
- getNumeratorTotal()
  : umms.core.model.score.NewRatioScore
  , umms.core.model.score.RatioScore
  , umms.core.model.score.WindowAllScore
- getNumExons()
  : umms.core.annotation.Gene
- getNumGC()
  : broad.core.primer3.Primer3PickingSummary
- getNumGCClampFailures()
  : broad.core.primer3.Primer3PickingSummary
- getNumHotMeltingTemp()
  : broad.core.primer3.Primer3PickingSummary
- getNumIsoformsPerSet()
  : broad.pda.annotation.Locus
- getNumLeft()
  : broad.core.math.CombinationGenerator
- getNumMarkers()
  : broad.pda.seq.alignment.StoredAlignmentStats
- getNumMispriming()
  : broad.core.primer3.Primer3PickingSummary
- getNumNs()
  : broad.core.primer3.Primer3PickingSummary
- getNumOk()
  : broad.core.primer3.Primer3PickingSummary
- getNumOligosTested()
  : broad.core.primer3.Primer3PickingSummary
- getNumOverlappingAnnotations()
  : umms.core.annotation.AnnotationList< T extends Annotation >
- getNumOverlappingExcludedRegions()
  : broad.core.primer3.Primer3PickingSummary
- getNumOverlappingTargets()
  : broad.core.primer3.Primer3PickingSummary
- getNumPolyX()
  : broad.core.primer3.Primer3PickingSummary
- getNumPossibleKmers()
  : broad.core.motif.SequenceMotif
- getNumReads()
  : umms.core.model.AlignmentModel.AlignmentCount
  , umms.core.model.JCSAlignmentModel.AlignmentCount
- getNumRows()
  : Jama.Matrix
- getNumSelfComplementarityAnywhere()
  : broad.core.primer3.Primer3PickingSummary
- getNumSeqQuality()
  : broad.core.primer3.Primer3PickingSummary
- getNumStartPosToErase()
  : broad.core.motif.PositionWeightMatrix
- getNumUniqueReads()
  : broad.pda.countreads.FastqLibraryStats
- getOffset()
  : broad.core.sequence.AbstractFastaParser
- getOneRandomSamplePermutation()
  : broad.pda.seq.protection.MultiSampleScanPeakCaller
- getOpen()
  : jaligner.Alignment
- getOppositeVertex()
  : broad.pda.seq.graph.ChromosomeWithBubblesJGraphT
- getOrCreatePairedEndFile()
  : umms.core.readers.PairedEndReader
- getOrdered()
  : broad.pda.geneexpression.clustering.Cluster
- getOrderedAssembly()
  : umms.core.scripture.statistics.ConnectDisconnectedTranscripts
- getOrderedListCDT()
  : broad.core.util.ParseGCTFile
- getOrientation()
  : broad.core.annotation.GenomicAnnotation
  , broad.core.annotation.LightweightGenomicAnnotation
  , broad.core.sequence.SequenceRegion
  , broad.core.sequence.TranscribedRegions
  , broad.pda.seq.graph.Path
  , umms.core.alignment.AbstractPairedEndAlignment
  , umms.core.annotation.Annotation
  , umms.core.annotation.BasicAnnotation
- getOrientedEnd()
  : broad.core.annotation.BasicGenomicAnnotation
  , broad.core.annotation.GenomicAnnotation
  , broad.core.sequence.SequenceRegion
  , umms.core.annotation.AbstractAnnotation
  , umms.core.annotation.Annotation
- getOrientedLastExon()
  : umms.core.annotation.Gene
- getOrientedStart()
  : broad.core.annotation.BasicGenomicAnnotation
  , broad.core.annotation.GenomicAnnotation
  , broad.core.sequence.SequenceRegion
  , umms.core.annotation.AbstractAnnotation
  , umms.core.annotation.Annotation
- getOriginalBamFile()
  : broad.pda.seq.clip.SampleData
  , broad.pda.seq.protection.SampleData
- getOriginalMValues()
  : broad.core.math.Loess
- getOriginalScore()
  : broad.core.primer3.HairpinKmer
- getOriginalSequence1()
  : jaligner.Alignment
- getOriginalSequence2()
  : jaligner.Alignment
- getOrphanNodes()
  : broad.pda.seq.graph.ChromosomeWithBubblesJGraphT
- getOrphanPaths()
  : broad.pda.seq.graph.ChromosomeWithBubblesJGraphT
  , umms.core.scripture.OrientedChromosomeTranscriptGraph
- getOrphanVertices()
  : umms.core.scripture.OrientedChromosomeTranscriptGraph
- getOutOrUnorientedEdges()
  : broad.pda.seq.graph.ChromosomeWithBubblesJGraphT
- getOutputFile()
  : umms.core.writers.PairedEndWriter
- getOverlap()
  : broad.core.annotation.BasicLightweightAnnotation
  , broad.core.annotation.LightweightGenomicAnnotation
  , broad.core.overlaputils.GeneSetIntersect
  , broad.core.primer3.HairpinKmer
  , broad.core.sequence.SequenceRegion
  , umms.core.annotation.Annotation
  , umms.core.annotation.BasicAnnotation
  , umms.core.annotation.Gene
- getOverlappers()
  : broad.core.annotation.AnnotationReader< T extends GenomicAnnotation >
  , broad.core.siphy.StationaryDistributionIO
  , broad.pda.annotation.BEDFileParser
  , broad.pda.datastructures.LocationAwareMatrix
  , umms.core.annotation.Gene
- getOverlappingAnnotationList()
  : umms.core.annotation.AnnotationList< T extends Annotation >
- getOverlappingAnnotations()
  : umms.core.annotation.AbstractAnnotationCollection< T extends Annotation >
  , umms.core.annotation.AnnotationCollection< T extends Annotation >
  , umms.core.annotation.AnnotationList< T extends Annotation >
  , umms.core.model.AlignmentModel
  , umms.core.model.JCSAlignmentModel
- getOverlappingGenes()
  : umms.core.scripture.BuildScriptureCoordinateSpace
- getOverlappingNodes()
  : broad.pda.seq.graph.Path
- getOverlappingReadCounts()
  : umms.core.model.AlignmentModel
  , umms.core.model.JCSAlignmentModel
- getOverlappingReadReplicateCounts()
  : umms.core.model.AlignmentModel
  , umms.core.model.JCSAlignmentModel
- getOverlappingReads()
  : umms.core.model.AlignmentModel
  , umms.core.model.JCSAlignmentModel
- getOverlappingRegion()
  : umms.core.coordinatesystem.CoordinateSpace
  , umms.core.coordinatesystem.GenomicSpace
  , umms.core.coordinatesystem.MaskedGenomicSpace
  , umms.core.coordinatesystem.TranscriptInGenomicSpace
  , umms.core.coordinatesystem.TranscriptomeSpace
- getOverlappingRegions()
  : broad.pda.seq.segmentation.AlignmentDataModel
  , broad.pda.seq.segmentation.GenericAlignmentDataModel
- getOverlappingSplicedReads()
  : umms.core.model.AlignmentModel
  , umms.core.model.JCSAlignmentModel
- getOverlappingVertices()
  : broad.pda.seq.graph.ChromosomeWithBubblesJGraphT
- getOverlapWithRandomizedPositions()
  : broad.core.overlaputils.GeneSetIntersect
- getP()
  : broad.core.math.MannWhitneyPermutations
  , broad.core.math.Wilcox2Sample
- getPair1Parser()
  : broad.pda.seq.fastq.FastqSplitterByBarcode
- getPair2Parser()
  : broad.pda.seq.fastq.FastqSplitterByBarcode
- getPairedData()
  : broad.pda.seq.segmentation.ContinuousDataAlignmentModel
- getPairedEndEdges()
  : broad.pda.seq.graph.Path
- getPairedEndFile()
  : umms.core.readers.PairedEndReader
- getParameters()
  : broad.core.siphy.EvolutionaryModel
  , broad.core.util.CLUtil
- getParents()
  : umms.core.utils.AnnotationUtils
- getPaths()
  : broad.pda.seq.graph.ChromosomeWithBubblesJGraphT
  , umms.core.scripture.BuildScriptureCoordinateSpace
  , umms.core.scripture.OrientedChromosomeTranscriptGraph
- getPeak()
  : broad.pda.seq.segmentation.AlignmentDataModel
  , broad.pda.seq.segmentation.AlignmentDataModelStats
  , broad.pda.seq.segmentation.ContinuousDataAlignmentModel
  , broad.pda.seq.segmentation.GenericAlignmentDataModel
  , umms.core.model.AlignmentModel
  , umms.core.model.JCSAlignmentModel
- getPeakBedFileName()
  : broad.pda.seq.protection.MultiSampleScanPeakCaller
- getPeakScores()
  : broad.pda.seq.rap.CollectAnnotationEnrichments
- getPercent3UTR()
  : umms.core.annotation.Gene
- getPercentCDS()
  : umms.core.annotation.Gene
- getPercentConsistent()
  : broad.pda.seq.graph.Path
- getPercentDuplicated()
  : broad.pda.countreads.FastqLibraryStats
- getPercentFeaturesWellAboveBackground()
  : broad.pda.geneexpression.agilent.AgilentArrayStats
- getPercentGC()
  : broad.core.primer3.HairpinKmer
- getPercentIdentity()
  : jaligner.Alignment
  , umms.core.annotation.PSLRecord
- getPercentOfValuesBetween()
  : broad.core.math.EmpiricalDistribution
- getPercentOfValuesLargerThan()
  : broad.core.math.EmpiricalDistribution
- getPercentVariantsCovered()
  : broad.core.primer3.HairpinKmer
- getPermNum()
  : broad.pda.gene.NeighborsNullModel
- getPermutationMatrix()
  : broad.pda.differentialExpression.DifferentialExpression
- getPermutedAnnotations()
  : umms.core.annotation.AnnotationCollection< T extends Annotation >
  , umms.core.annotation.AnnotationList< T extends Annotation >
  , umms.core.model.AlignmentModel
  , umms.core.model.JCSAlignmentModel
- getPi()
  : broad.core.siphy.EvolutionaryModel
- getPIDToName()
  : broad.core.datastructures.MatrixWithHeaders
- getPivot()
  : Jama.LUDecomposition
- getPlateName()
  : broad.pda.geneexpression.ExpressionExperimentInfo
- getPoissonLambda()
  : broad.pda.seq.protection.TwoSampleScanSkellamPeakCaller
- getPosition()
  : broad.core.siphy.Fit
- getPositionAtCoordinate()
  : umms.core.annotation.CompoundInterval
- getPositionAtReferenceCoordinate()
  : broad.core.sequence.SequenceRegion
  , umms.core.annotation.AbstractAnnotation
  , umms.core.annotation.Annotation
  , umms.core.annotation.BasicAnnotation
- getPositionCountList()
  : umms.core.model.AlignmentModel
- getPositionCountMap()
  : umms.core.coordinatesystem.TranscriptomeSpace
- getPreferredFileExtension()
  : broad.core.multiplealignment.FastaMultipleAlignmentIO
  , broad.core.multiplealignment.MAFIO
  , broad.core.multiplealignment.MultipleAlignmentIO
  , broad.core.multiplealignment.PhylipInterleavedMultipleAlignmentIO
  , broad.core.multiplealignment.PhylipSequencialMultipleAlignmentIO
- getPrimerPickingSummary()
  : broad.core.primer3.Primer3PrimerPickingResult
- getPrimers()
  : broad.core.primer3.Primer3PrimerPickingResult
- getPriorClosest()
  : broad.pda.datastructures.LocationAwareMatrix
- getProbability()
  : broad.core.math.EmpiricalDistribution
  , broad.core.motif.PositionWeightColumn
- getProbename()
  : broad.pda.geneexpression.agilent.AgilentProbe
- getProcessedSignal()
  : broad.pda.geneexpression.agilent.AgilentProbe
- getPromoter()
  : umms.core.annotation.Gene
- getPromoters()
  : broad.pda.annotation.BEDFileParser
- getPVal()
  : broad.core.siphy.EvolutionaryModel
- getPValue()
  : broad.core.math.EmpiricalDistribution
- getPvalue()
  : broad.core.math.MannWhitney
- getPValue()
  : broad.pda.enrichment.EnrichmentMetric
  , umms.core.annotation.Gene
- getPvalue()
  : umms.core.model.score.BasicScanStatisticScore
  , umms.core.model.score.BinomialEnrichmentScore
  , umms.core.model.score.CountScore
  , umms.core.model.score.MultiScore
  , umms.core.model.score.PoissonEnrichmentScore
  , umms.core.model.score.ScanStatisticScore
- getPValue2()
  : broad.core.math.EmpiricalDistribution
- getPWM()
  : broad.core.motif.meme.MemeMotif
- getPWMiterator()
  : broad.core.siphy.ConservedPWMScanner
- getQ()
  : Jama.QRDecomposition
- getQualityBases()
  : broad.core.sequence.FastaQualityBinaryIO
- getQuantileOfBins()
  : broad.core.math.EmpiricalDistribution
- getR()
  : Jama.QRDecomposition
- getRandomAnnotation()
  : umms.core.coordinatesystem.TranscriptomeSpace.GeneTree
- getRandomSets()
  : broad.pda.gene.transcriptsNullModel
- getRankedList()
  : broad.projection.gsa.GeneSetEnrichment
- getRateMatrix()
  : broad.core.siphy.EvolutionaryModel
  , broad.core.siphy.EvolutionaryModelParameters
- getRatio()
  : umms.core.model.score.NewRatioScore
  , umms.core.model.score.RatioScore
  , umms.core.model.score.WindowAllScore
- getRawDensity()
  : broad.core.math.EmpiricalDistribution
- getRead()
  : umms.core.model.AlignmentModel.AlignmentCount
  , umms.core.model.JCSAlignmentModel.AlignmentCount
- getRead1Trim3p()
  : broad.pda.seq.fastq.FastqSplitterByBarcode
- getRead1Trim5p()
  : broad.pda.seq.fastq.FastqSplitterByBarcode
- getRead2Trim3p()
  : broad.pda.seq.fastq.FastqSplitterByBarcode
- getRead2Trim5p()
  : broad.pda.seq.fastq.FastqSplitterByBarcode
- getReadAlignmentBlocks()
  : umms.core.alignment.AbstractPairedEndAlignment
  , umms.core.alignment.Alignment
  , umms.core.alignment.SingleEndAlignment
- getReadAlignments()
  : umms.core.alignment.AbstractPairedEndAlignment
  , umms.core.alignment.Alignment
  , umms.core.alignment.SingleEndAlignment
- getReader()
  : broad.pda.samtools.SAMPairedEndFileReader
- getReadIterator()
  : broad.pda.seq.segmentation.AlignmentDataModel
  , broad.pda.seq.segmentation.AlignmentDataModelStats
  , broad.pda.seq.segmentation.GenericAlignmentDataModel
  , umms.core.model.AlignmentModel
  , umms.core.model.JCSAlignmentModel
- getReadLength()
  : umms.core.alignment.SingleEndAlignment
- getReadMates()
  : umms.core.alignment.AbstractPairedEndAlignment
  , umms.core.alignment.Alignment
  , umms.core.alignment.SingleEndAlignment
- getReadName()
  : broad.pda.samtools.FilterFastqReads
  , umms.core.alignment.AbstractPairedEndAlignment
  , umms.core.alignment.Alignment
  , umms.core.alignment.SingleEndAlignment
- getReads()
  : broad.core.datastructures.JCSCache
- getReadSequence()
  : umms.core.alignment.AbstractPairedEndAlignment
  , umms.core.alignment.Alignment
  , umms.core.alignment.SingleEndAlignment
- getReadSizeDistribution()
  : umms.core.model.AlignmentModel
  , umms.core.model.JCSAlignmentModel
- getReadString()
  : umms.core.alignment.SingleEndAlignment.WrapSamRecord
- getRealEigenvalues()
  : Jama.EigenvalueDecomposition
- getReasonForUnsafe()
  : broad.pda.seq.alignment.StoredAlignmentStats
- getReducedRepresentation()
  : broad.pda.feature.genome.DirectoryInstalledGenomeAssembly
- getReference()
  : broad.core.multiplealignment.MultipleAlignment
- getReferenceAnnotation()
  : umms.core.coordinatesystem.CoordinateSpace
  , umms.core.coordinatesystem.GenomicSpace
  , umms.core.coordinatesystem.MaskedGenomicSpace
  , umms.core.coordinatesystem.TranscriptInGenomicSpace
  , umms.core.coordinatesystem.TranscriptomeSpace
- getReferenceAnnotations()
  : umms.core.coordinatesystem.CoordinateSpace
  , umms.core.coordinatesystem.GenomicSpace
  , umms.core.coordinatesystem.MaskedGenomicSpace
  , umms.core.coordinatesystem.TranscriptInGenomicSpace
  , umms.core.coordinatesystem.TranscriptomeSpace
- getReferenceCoordinateAtPosition()
  : broad.core.sequence.SequenceRegion
  , umms.core.annotation.AbstractAnnotation
  , umms.core.annotation.Annotation
  , umms.core.annotation.BasicAnnotation
- getReferenceEnd()
  : broad.core.multiplealignment.MAFAlignment
  , broad.core.multiplealignment.MultipleAlignment
- getReferenceId()
  : broad.core.multiplealignment.MultipleAlignment
- getReferenceName()
  : broad.core.sequence.SequenceRegion
  , umms.core.annotation.Annotation
  , umms.core.annotation.BasicAnnotation
- getReferenceNames()
  : umms.core.coordinatesystem.CoordinateSpace
  , umms.core.coordinatesystem.GenomicSpace
  , umms.core.coordinatesystem.MaskedGenomicSpace
  , umms.core.coordinatesystem.TranscriptInGenomicSpace
  , umms.core.coordinatesystem.TranscriptomeSpace
- getReferenceStart()
  : broad.core.multiplealignment.MAFAlignment
  , broad.core.multiplealignment.MultipleAlignment
- getReferenceTranscript()
  : broad.pda.annotation.Locus
- getRefseqGeneName()
  : broad.core.sequence.Sequence
- getRefseqRnaClassName()
  : broad.core.sequence.Sequence
- getRefseqSpeciesName()
  : broad.core.sequence.Sequence
- getRefSequenceCounts()
  : umms.core.model.AlignmentModel
  , umms.core.model.JCSAlignmentModel
- getRefSequenceLambda()
  : umms.core.annotation.AnnotationCollection< T extends Annotation >
  , umms.core.annotation.AnnotationList< T extends Annotation >
  , umms.core.model.AlignmentModel
  , umms.core.model.JCSAlignmentModel
- getRefSequenceLength()
  : umms.core.model.AlignmentModel
  , umms.core.model.JCSAlignmentModel
- getRefSequenceLengths()
  : umms.core.model.AlignmentModel
  , umms.core.model.JCSAlignmentModel
  , umms.core.readers.PairedEndReader
- getRegion()
  : broad.core.sequence.Sequence
  , broad.core.sequence.SequenceRegion
  , broad.core.sequence.SequenceRegionWindow
  , broad.pda.feature.genome.Chromosome
  , broad.pda.rnai.RNAiGeneAnnotation
  , umms.core.coordinatesystem.TranscriptomeSpace.GeneTree
- getRegionDenominatorRPKM()
  : umms.core.model.score.NewRatioScore
  , umms.core.model.score.RatioScore
  , umms.core.model.score.WindowAllScore
- getRegionEnd()
  : broad.core.sequence.SequenceRegion
- getRegionLength()
  : umms.core.model.score.BasicScanStatisticScore
  , umms.core.model.score.BinomialEnrichmentScore
  , umms.core.model.score.MultiScore
  , umms.core.model.score.PoissonEnrichmentScore
  , umms.core.model.score.ScanStatisticScore
- getRegionNumeratorRPKM()
  : umms.core.model.score.NewRatioScore
  , umms.core.model.score.RatioScore
  , umms.core.model.score.WindowAllScore
- getRegionRatio()
  : umms.core.model.score.NewRatioScore
  , umms.core.model.score.RatioScore
  , umms.core.model.score.WindowAllScore
- getRegions()
  : broad.core.sequence.Sequence
  , broad.pda.feature.genome.Chromosome
  , broad.pda.seq.rap.GenomeCommandLineProgram
- getRegionSet()
  : broad.pda.seq.rap.GenomeCommandLineProgram
- getRegionsMap()
  : broad.pda.seq.rap.GenomeCommandLineProgram
- getRegionStart()
  : broad.core.sequence.SequenceRegion
- getRegionTotal()
  : umms.core.model.score.CountScore
- getRelationship()
  : broad.core.datastructures.Interval
- getRelativeGenomeLength()
  : broad.pda.seq.graph.ChromosomeWithBubblesJGraphT
  , broad.pda.seq.segmentation.GenomeWithGaps2
- getRelativePositions()
  : broad.core.primer3.HairpinKmer
- getRelativeWindow()
  : broad.pda.seq.segmentation.GenomeWithGaps2
- getRepeatClass()
  : broad.core.annotation.RepeatMaskerAnnotation
- getRepeatFamily()
  : broad.core.annotation.RepeatMaskerAnnotation
- getRepeatFile()
  : broad.pda.feature.genome.DirectoryInstalledGenomeAssembly
- getRepeatName()
  : broad.core.annotation.RepeatMaskerAnnotation
- getREST()
  : broad.pda.geneexpression.ScoreDiffGenes
- getResults()
  : broad.pda.differentialExpression.RunDifferentialExpression
- getReverseStrand()
  : umms.core.annotation.Annotation.Strand
- getReverseTargets()
  : umms.core.alignment.SmatchLike
- getRgb()
  : broad.core.annotation.BED
- getRight()
  : broad.pda.geneexpression.clustering.Cluster
- getRightHighInfoStart()
  : broad.core.motif.PositionWeightMatrix
- getRightNeighbor()
  : broad.pda.gene.NeighborAnalysis
- getRoot()
  : broad.core.datastructures.GenericRootedTree< T >
- getRow()
  : broad.core.datastructures.MatrixWithHeaders
  , jaligner.Cell
  , Jama.Matrix
- getRowAnnotations()
  : broad.pda.datastructures.LocationAwareMatrix
- getRowDescriptions()
  : broad.core.datastructures.MatrixWithHeaders
- getRowDimension()
  : Jama.Matrix
- getRowLocationMap()
  : broad.pda.datastructures.LocationAwareMatrix
- getRowName()
  : broad.core.datastructures.MatrixWithHeaders
- getRowNames()
  : broad.core.datastructures.MatrixWithHeaders
- getRownum()
  : broad.pda.geneexpression.agilent.AgilentProbe
- getRowPackedCopy()
  : Jama.Matrix
- getRowPosition()
  : broad.pda.datastructures.LocationAwareMatrix
- getRowTree()
  : broad.pda.datastructures.LocationAwareMatrix
- getRPK()
  : umms.core.model.score.CountScore
- getRPKM()
  : broad.pda.seq.segmentation.GeneScore
  , umms.core.annotation.Gene
  , umms.core.model.score.CountScore
- getRPKMConstant()
  : broad.pda.seq.graph.ChromosomeWithBubblesJGraphT
  , broad.pda.seq.segmentation.AlignmentDataModelStats
- getRS8Score()
  : broad.core.primer3.HairpinKmer
- getS()
  : Jama.SingularValueDecomposition
- getSAMEnd()
  : broad.core.sequence.SequenceRegion
  , umms.core.annotation.AbstractAnnotation
  , umms.core.annotation.Annotation
- getSamHeader()
  : broad.pda.seq.segmentation.GenericAlignmentDataModel
- getSampleCoordSpace()
  : umms.core.model.score.MultiScore
- getSampleCount()
  : umms.core.model.score.BinomialEnrichmentScore
  , umms.core.model.score.MultiScore
  , umms.core.model.score.PoissonEnrichmentScore
- getSampleLambda()
  : umms.core.model.score.PoissonEnrichmentScore
- getSampleName()
  : broad.pda.seq.clip.SampleData
  , broad.pda.seq.protection.SampleData
- getSampleRegionCount()
  : umms.core.model.score.BinomialEnrichmentScore
  , umms.core.model.score.MultiScore
- getSampleRegionTotal()
  : umms.core.model.score.PoissonEnrichmentScore
- getSampleTotal()
  : umms.core.model.score.PoissonEnrichmentScore
- getSampleType()
  : broad.pda.geneexpression.ExpressionExperimentInfo
- getSAMRecordIterator()
  : broad.pda.samtools.SAMPairedEndFileReader
- getSAMStart()
  : broad.core.sequence.SequenceRegion
  , umms.core.annotation.AbstractAnnotation
  , umms.core.annotation.Annotation
- getSamString()
  : broad.pda.samtools.SAMFragmentWriter
- getSAMString()
  : umms.core.annotation.Gene
- getSaturationSignalValue()
  : broad.pda.geneexpression.agilent.AgilentArrayStats
- getScaledWindows()
  : broad.core.siphy.TreeScalerIO
- getScanDistribution()
  : broad.core.math.PermutationDistribution
- getScanPvalFragmentEndsMaxPositionAllRegionsBam()
  : broad.pda.seq.clip.Pileup
- getScanPvalFragmentEndsMaxPositionBam()
  : broad.pda.seq.clip.Pileup
- getScanPvalsFragmentEndPileupsAllRegionsWig()
  : broad.pda.seq.clip.Pileup
- getScanPvalsFragmentEndPileupsWig()
  : broad.pda.seq.clip.Pileup
- getScanPvalue()
  : broad.core.math.PermutationDistribution
  , broad.pda.seq.segmentation.GeneScore
  , umms.core.model.score.BasicScanStatisticScore
  , umms.core.model.score.ScanStatisticScore
- getScore()
  : broad.core.annotation.BasicLightweightAnnotation
  , broad.core.annotation.Feature
  , broad.core.annotation.LightweightGenomicAnnotation
  , broad.core.multiplealignment.MultipleAlignment
  , broad.core.sequence.SequenceRegion
  , broad.core.sequence.SequenceRegionWindow
  , broad.pda.datastructures.Alignments
  , broad.pda.geneexpression.clustering.Cluster
  , broad.pda.seq.graph.Path
  , broad.pda.seq.rap.SkellamScore
  , jaligner.Alignment
  , jaligner.Cell
  , jaligner.matrix.Matrix
  , umms.core.annotation.Annotation
  , umms.core.annotation.BasicAnnotation
  , umms.core.model.score.AbstractListScore
  , umms.core.model.score.CountScore
  , umms.core.model.score.EndInWindowCountScore
  , umms.core.model.score.NewRatioScore
  , umms.core.model.score.RatioScore
  , umms.core.model.score.StartInWindowCountScore
  , umms.core.model.score.SumScore
  , umms.core.model.score.WindowAllScore
  , umms.core.model.score.WindowScore
- getScoredExons()
  : broad.pda.gene.GeneWithIsoforms
- getScorePerAlignmentFromCache()
  : broad.pda.seq.segmentation.AlignmentDataModel
  , broad.pda.seq.segmentation.AlignmentDataModelStats
  , broad.pda.seq.segmentation.GenericAlignmentDataModel
- getScores()
  : broad.pda.datastructures.Alignments
  , jaligner.matrix.Matrix
  , umms.core.model.score.AbstractListScore
  , umms.core.model.score.BasicScanStatisticScore
  , umms.core.model.score.ScanStatisticScore
- getScoreSum()
  : broad.core.annotation.AnnotationReader< T extends GenomicAnnotation >
- getScoreWithNoTerminalGaps()
  : jaligner.Alignment
- getSecondGene()
  : umms.core.alignment.FeatureSequenceAlignment.UnorderedGenePair
- getSecondMate()
  : umms.core.alignment.AbstractPairedEndAlignment
- getSentinel()
  : broad.core.datastructures.IntervalTree< V >
- getSequence()
  : broad.core.annotation.BasicGenomicAnnotation
  , broad.core.annotation.GenomicAnnotation
  , broad.core.sequence.SequenceRegion
  , broad.pda.datastructures.Alignments
  , broad.pda.feature.genome.Chromosome
  , broad.pda.rnai.RNAiGeneAnnotation
  , broad.pda.seq.fastq.FastqSequence
  , jaligner.Sequence
  , umms.core.annotation.Gene
- getSequence1()
  : jaligner.Alignment
- getSequence2()
  : jaligner.Alignment
- getSequenceAnnotationMap()
  : broad.core.annotation.AnnotationReader< T extends GenomicAnnotation >
- getSequenceBases()
  : broad.core.sequence.Sequence
- getSequenceBuilder()
  : broad.core.sequence.Sequence
- getSequenceDir()
  : broad.pda.feature.genome.DirectoryInstalledGenomeAssembly
- getSequenceForGene()
  : broad.pda.rnai.ExtractSequence
- getSequenceName()
  : broad.core.annotation.GFF
- getSequenceNames()
  : broad.core.sequence.FastaSequenceIO
  , broad.pda.samtools.SAMPairedEndFileReader
  , umms.core.readers.PairedEndReader
- getSequenceObject()
  : umms.core.annotation.Gene
- getSequences()
  : broad.pda.seq.fastq.FastqParser
- getSequenceUnoriented()
  : broad.pda.rnai.ExtractSequence
- getSetIsoforms()
  : broad.pda.annotation.Locus
- getShortArm()
  : broad.pda.feature.genome.Chromosome
- getShuffledPositions()
  : broad.pda.seq.clip.Pileup
- getShuffledPositionsAllRegions()
  : broad.pda.seq.clip.Pileup
- getSignificantGeneSets()
  : broad.pda.geneexpression.PaGEJava
- getSilhouette()
  : broad.pda.geneexpression.clustering.Kmeans
- getSimilarity()
  : jaligner.Alignment
- getSingleReadsSkipped()
  : broad.pda.samtools.PairedEndReadIterator
- getSingleSampleScanPeaks()
  : broad.pda.seq.protection.MultiSampleScanPeakCaller
- getSingularValues()
  : Jama.SingularValueDecomposition
- getSinkVertices()
  : broad.pda.seq.graph.ChromosomeWithBubblesJGraphT
  , umms.core.scripture.OrientedChromosomeTranscriptGraph
- getSites()
  : broad.core.motif.meme.MemeMotif
- getSize()
  : broad.core.sequence.SequenceRegion
  , broad.pda.seq.graph.Path
  , umms.core.annotation.AbstractAnnotation
  , umms.core.annotation.Annotation
  , umms.core.annotation.Gene
  , umms.core.coordinatesystem.CoordinateSpace
  , umms.core.coordinatesystem.GenomicSpace
  , umms.core.coordinatesystem.MaskedGenomicSpace
  , umms.core.coordinatesystem.TranscriptInGenomicSpace
  , umms.core.coordinatesystem.TranscriptomeSpace
  , umms.core.feature.GeneWindow
- getSizesFromSAM()
  : broad.pda.seq.alignment.AlignmentUtils
- getSkellamPvalue()
  : broad.pda.seq.protection.TwoSampleScanSkellamPeakCaller
- getSkellamPValue()
  : broad.pda.seq.rap.SkellamScore
  , umms.core.model.score.WindowAllScore
- getSlider()
  : broad.core.sequence.Sequence
  , broad.core.sequence.SequenceRegion
  , broad.core.sequence.WindowSlider
- getSoftmaskedRegions()
  : broad.core.sequence.Sequence
- getSortedAndUniqueExons()
  : umms.core.annotation.Gene
- getSortedNodes()
  : broad.pda.seq.graph.Path
- getSource()
  : broad.core.annotation.GFF
- getSourceAnnotations()
  : umms.core.feature.GeneWindow
  , umms.core.feature.GenomeWindow
  , umms.core.feature.Window
- getSourceVertices()
  : broad.pda.seq.graph.ChromosomeWithBubblesJGraphT
  , umms.core.scripture.OrientedChromosomeTranscriptGraph
- getSpan()
  : umms.core.annotation.CompoundInterval
- getSpikeInDetectionLimit()
  : broad.pda.geneexpression.agilent.AgilentArrayStats
- getSpliceConnections()
  : broad.core.sequence.SequenceRegion
  , umms.core.alignment.Alignment
  , umms.core.alignment.SingleEndAlignment
  , umms.core.annotation.Annotation
  , umms.core.annotation.BasicAnnotation
- getSplicedCount()
  : umms.core.model.AlignmentModel
  , umms.core.model.JCSAlignmentModel
- getSplicedReadExons()
  : broad.pda.seq.segmentation.AlignmentDataModel
  , broad.pda.seq.segmentation.GenericAlignmentDataModel
- getSplicedReads()
  : broad.pda.seq.segmentation.AlignmentDataModel
  , broad.pda.seq.segmentation.AlignmentDataModelStats
  , broad.pda.seq.segmentation.GenericAlignmentDataModel
- getSpliceJunctionCoordinates()
  : umms.core.annotation.Gene
- getSpliceJunctionCoordinatesTree()
  : umms.core.annotation.Gene
- getSpliceWeight()
  : broad.pda.seq.graph.ChromosomeWithBubblesJGraphT
  , broad.pda.seq.graph.Path
- getSpliceWeightFactor()
  : broad.pda.seq.segmentation.AlignmentDataModel
  , broad.pda.seq.segmentation.AlignmentDataModelStats
  , broad.pda.seq.segmentation.GenericAlignmentDataModel
- getStandardDeviation()
  : broad.core.math.EmpiricalDistribution
  , umms.core.model.score.AbstractListScore
- getStart()
  : broad.core.annotation.LightweightGenomicAnnotation
  , broad.core.datastructures.Interval
  , broad.core.sequence.SequenceRegion
  , broad.pda.seq.graph.ChromosomeWithBubblesJGraphT
  , broad.pda.seq.graph.Path
  , umms.core.alignment.AbstractPairedEndAlignment
  , umms.core.alignment.Alignment
  , umms.core.annotation.Annotation
  , umms.core.annotation.BasicAnnotation
  , umms.core.annotation.CompoundInterval
- getStart1()
  : jaligner.Alignment
- getStart2()
  : jaligner.Alignment
- getStartCodon()
  : umms.core.annotation.Gene
- getStartVertex()
  : broad.pda.seq.graph.Path
- getState()
  : broad.core.hmm.MarkovModel< T >
- getStates()
  : broad.core.hmm.MarkovModel< T >
- getStateTransitionMatrix()
  : broad.core.hmm.MarkovModel< T >
- getStatistic()
  : broad.pda.enrichment.EnrichmentMetric
- getStepSize()
  : broad.pda.seq.protection.SampleData
- getStrand()
  : broad.core.annotation.GFF
  , broad.core.sequence.SequenceRegion
  , umms.core.annotation.AbstractAnnotation
  , umms.core.annotation.Annotation
  , umms.core.model.AlignmentModel
- getString()
  : umms.core.annotation.Gene
- getStringArg()
  : broad.core.parser.CommandLineParser
- getStringArray()
  : broad.core.parser.StringParser
- getStringListArg()
  : broad.core.parser.CommandLineParser
- getSubAlignment()
  : broad.core.multiplealignment.MAFAlignment
  , broad.core.multiplealignment.MultipleAlignment
- getSubAnnotationFromEnd()
  : umms.esat.ESAT
  , umms.esat.ESATDistanceCalculator
- getSubAnnotationFromStart()
  : umms.esat.ESAT
  , umms.esat.ESATDistanceCalculator
- getSubAnnotationMidpointWithinAnnotation()
  : umms.core.utils.AnnotationUtils
- getSubclusters()
  : broad.pda.geneexpression.clustering.Cluster
- getSubmethod()
  : broad.pda.enrichment.EnrichmentMetric
- getSubSequence()
  : broad.core.sequence.Sequence
- getSubsequence()
  : broad.core.sequence.Sequence
- getSubSequence()
  : broad.core.sequence.Sequence
- getSubsequence()
  : broad.core.sequence.Sequence
- getSubSequence()
  : broad.core.sequence.Sequence
- getSum()
  : broad.pda.seq.segmentation.AlignmentDataModelStats
  , broad.pda.seq.segmentation.ContinuousDataAlignmentModel
  , umms.core.model.score.AbstractListScore
- getSummary()
  : jaligner.Alignment
- getSymbol()
  : broad.pda.feature.genome.Chromosome
- getTermGenes()
  : broad.pda.gene.functionalAnnotation
- getTestStatistic()
  : broad.core.math.FisherCombinedProbabilityTest
- getTestStatisticMatrix()
  : broad.pda.differentialExpression.DifferentialExpression
- getThickEnd()
  : broad.core.annotation.BED
- getThickStart()
  : broad.core.annotation.BED
- getThreePrimeBases()
  : broad.core.annotation.BasicGenomicAnnotation
  , broad.core.annotation.GenomicAnnotation
  , broad.core.sequence.SequenceRegion
- getTimePoint()
  : broad.pda.geneexpression.ExpressionExperimentInfo
- getTokens()
  : broad.core.parser.StringParser
- getTotal()
  : broad.core.math.CombinationGenerator
  , umms.core.model.score.CountScore
- getTotalCoverage()
  : umms.core.annotation.AbstractAnnotationCollection< T extends Annotation >
  , umms.core.annotation.AnnotationCollection< T extends Annotation >
- getTotalDistanceFromNode()
  : broad.core.siphy.TreeScaler
- getTotalInSources()
  : broad.core.siphy.TreeScalerIO
- getTotalNumberOffeatures()
  : broad.pda.geneexpression.agilent.AgilentArrayStats
- getTotalNumberOfMappedReads()
  : broad.pda.seq.segmentation.GenericAlignmentDataModel
- getTotalNumberOfMappedStrandedReads()
  : broad.pda.seq.segmentation.GenericAlignmentDataModel
- getTotalNumberOfReads()
  : broad.pda.seq.segmentation.GenericAlignmentDataModel
- getTotalNumberOfStrandedReads()
  : broad.pda.seq.segmentation.AlignmentDataModel
  , broad.pda.seq.segmentation.GenericAlignmentDataModel
- getTotalObservations()
  : broad.core.math.EmpiricalDistribution
- getTotalReads()
  : broad.pda.countreads.FastqLibraryStats
  , broad.pda.seq.segmentation.AlignmentDataModelStats
- getTrackInfo()
  : broad.core.annotation.AnnotationReader< T extends GenomicAnnotation >
- getTranscribedBase()
  : broad.core.sequence.TranscribedRegions
- getTranscribedBaseAtBeginningPosition()
  : broad.core.sequence.TranscribedSequenceComposition
- getTranscriptLength()
  : umms.core.annotation.Gene
- getTranscriptName()
  : broad.pda.rnai.RNAiGeneAnnotation
- getTranscriptomeSpace()
  : broad.pda.seq.rap.rna.TranscriptomeCommandLineProgram
  , umms.core.coordinatesystem.TranscriptomeSpace
- getTranscripts()
  : broad.core.primer3.HairpinKmer
- getTranscriptSequence()
  : broad.pda.rnai.RNAiGeneAnnotation
- getTree()
  : broad.core.siphy.EvolutionaryModel
  , broad.core.siphy.EvolutionaryModelParameters
- getType()
  : jaligner.Sequence
- getU()
  : Jama.LUDecomposition
  , Jama.SingularValueDecomposition
- getUn()
  : broad.pda.feature.genome.DirectoryInstalledGenomeAssembly
- getUngappedReferenceIslands()
  : broad.core.multiplealignment.MultipleAlignment
- getUngappedSequenceReferenceIslands()
  : broad.core.multiplealignment.MultipleAlignment
- getUngappedSize()
  : broad.pda.feature.genome.Chromosome
- getUniqueNeighbors()
  : broad.pda.gene.NeighborsNullModel
- getUniqueRepeatClasses()
  : broad.core.annotation.RepeatMaskerAnnotationReader
- getUniqueRepeatFamilies()
  : broad.core.annotation.RepeatMaskerAnnotationReader
- getUniqueRepeatNames()
  : broad.core.annotation.RepeatMaskerAnnotationReader
- getUnmaskedLength()
  : umms.core.coordinatesystem.GenomicSpace
  , umms.core.coordinatesystem.TranscriptInGenomicSpace
- getUnqComaptibleBetweenAny2Sets()
  : broad.pda.annotation.Locus
- getUnqComaptibleBetweenSubsets()
  : broad.pda.annotation.Locus
- getUserDirectory()
  : jaligner.util.Commons
- getUtr3Counts()
  : broad.pda.seq.segmentation.GeneCounts
- getUtr5Counts()
  : broad.pda.seq.segmentation.GeneCounts
- getV()
  : Jama.EigenvalueDecomposition
  , Jama.SingularValueDecomposition
- getValidOutgoingEdges()
  : broad.pda.seq.graph.ChromosomeWithBubblesJGraphT
- getValue1()
  : broad.core.datastructures.Pair< T1 >
- getValue2()
  : broad.core.datastructures.Pair< T1 >
- getValues()
  : broad.core.annotation.GFF
  , broad.core.datastructures.MatrixWithHeaders
- getVectorEncodedSequence()
  : broad.core.sequence.Sequence
- getWeight()
  : broad.core.motif.PositionWeightColumn
  , broad.pda.seq.graph.Path
  , broad.pda.seq.segmentation.GenericAlignmentDataModel
  , umms.core.alignment.AbstractPairedEndAlignment
  , umms.core.alignment.Alignment
  , umms.core.alignment.SingleEndAlignment
- getWidth()
  : broad.core.motif.meme.MemeMotif
- getWidths()
  : broad.pda.seq.segmentation.ContinuousDataAlignmentModel
- getWindowCount()
  : broad.pda.seq.protection.SampleData
- getWindowIterator()
  : umms.core.coordinatesystem.CoordinateSpace
  , umms.core.coordinatesystem.GenomicSpace
  , umms.core.coordinatesystem.MaskedGenomicSpace
  , umms.core.coordinatesystem.TranscriptInGenomicSpace
  , umms.core.coordinatesystem.TranscriptomeSpace
- getWindowLength()
  : umms.core.model.score.BasicScanStatisticScore
- getWindowProcessor()
  : broad.pda.seq.rap.GenomeScoringProgram
  , broad.pda.seq.rap.rna.TranscriptomeScoringProgram
- getWindows()
  : umms.core.annotation.Gene
  , umms.core.feature.GeneWindow
  , umms.core.feature.GenomeWindow
  , umms.core.feature.Window
- getWindowScoreIterator()
  : broad.pda.seq.rap.SlideAndCalculate
- getWindowScores()
  : broad.pda.seq.protection.SampleData
- getWindowSize()
  : broad.pda.seq.protection.SampleData
- getX()
  : broad.pda.feature.genome.DirectoryInstalledGenomeAssembly
- getZ()
  : broad.core.math.MannWhitney
  , broad.core.math.Wilcox2Sample
- getZscore()
  : broad.core.math.EmpiricalDistribution
- GFF()
  : broad.core.annotation.GFF
- gffFactory
  : broad.core.annotation.AnnotationFactoryFactory
- GFFReader()
  : broad.core.annotation.GFFReader
- GlobalSpliceFilter()
  : broad.pda.seq.segmentation.GlobalSpliceFilter
- GlobalStats()
  : broad.pda.geneexpression.GlobalStats
- gotWindowScoresFromFile()
  : broad.pda.seq.protection.SampleData
- GrabProbes()
  : broad.pda.differentialExpression.GrabProbes
- GSAProjection()
  : broad.pda.differentialExpression.GSAProjection
- GSAScoring()
  : broad.projection.gsa.GSAScoring
- GTFFileParser()
  : broad.pda.annotation.GTFFileParser
- GTFWrite()
  : broad.pda.annotation.GTFFileParser


---

Generated on Fri Aug 15 2014 15:11:16 for ESAT by  

 1.8.7
